# Recycled PET/Sepiolite Composite Adsorbent for Methylene Blue Removal: Formulation, RSM Optimisation, Reusability, and Adsorption Mechanism

**DOI:** 10.3390/ijms27198841

**Published:** 2026-10-03

**Authors:** Suria Fatin Mohd Din, Zahid Iqbal Khan, Mohammed E. Ali Mohsin, Suleiman Mousa, Norhayani Othman, Mohammad Yousaf Younes, Zurina Mohamad, SK Safdar Hossain

**Affiliations:** 1 Faculty of Chemical and Energy Engineering, Universiti Teknologi Malaysia (UTM), Johor Bahru 81310, Johor, Malaysianorhayani@utm.my (N.O.);; 2 Interdisciplinary Research Centre for Advanced Materials, King Fahd University of Petroleum & Minerals, Dhahran 31261, Saudi Arabia; 3 Department of Chemical Engineering, College of Engineering, King Faisal University, P.O. Box 400, Al Ahsa 31982, Saudi Arabia

**Keywords:** recycled PET, sepiolite, methylene blue, adsorption, response surface methodology, Box–Behnken design, wastewater treatment

## Abstract

This study explores the valorisation of post-industrial recycled polyethylene terephthalate (rPET) via incorporation of sepiolite clay to develop a composite adsorbent for methylene blue (MB) dye removal. The composite formulation was evaluated by varying the rPET concentration, TFA:DCM solvent ratio, sepiolite content, and blending time. The selected formulation had an average particle size of 267.10 nm, a specific surface area of 0.67 m^2^/g, and a pore volume of 0.003925 cm^3^/g. Adsorption conditions were then optimised using a five-factor, three-level Box–Behnken design, considering contact time, MB concentration, adsorbent dosage, pH, and temperature. The resulting quadratic model was statistically significant (F = 114.18, *p* < 0.0001), with R^2^ = 0.9892, adjusted R^2^ = 0.9805, and predicted R^2^ = 0.9568; however, the lack-of-fit was also significant (F = 90.90, *p* < 0.0001), indicating that model predictions should be interpreted within the investigated factor ranges. At the selected conditions of 62.5 min, 25.5 mg/L MB, 2.55 g/50 mL adsorbent, pH 7, and 55 °C, the model predicted an MB removal efficiency of 99.03%. The adsorbent retained at least 93% reusability efficiency after six adsorption–washing–reuse cycles. The kinetic data were described more closely by the pseudo-second-order model, whereas the Freundlich model provided a better representation of the equilibrium data (R^2^ = 0.9976). Thermodynamic results indicated that adsorption was favourable and exothermic within the investigated temperature range. Overall, the results show that post-industrial PET can be converted into a value-added sepiolite-containing adsorbent for MB removal.

## 1. Introduction

Synthetic dyes are extensively used in industries such as textiles, printing, paper, leather, and pharmaceuticals, and their release into aquatic systems continues to raise serious environmental concerns [1]. Even small amounts of dye in wastewater can produce noticeable colour, reduce light penetration, interfere with photosynthesis, and adversely affect aquatic ecosystems while also increasing chemical oxygen demand. Methylene blue (MB) is commonly selected as a representative cationic dye in adsorption studies because it is highly soluble in water, strongly coloured, aromatic in structure, and relatively persistent in aqueous systems [2,3]. MB has several industrial and laboratory applications; however, its uncontrolled release into water bodies can cause ecological and health-related issues, which makes its removal from wastewater an important research priority [1]. Textile dye wastewater is recognised worldwide as a major contributor to industrial water pollution, and its complex composition can make effective treatment challenging when relying on conventional processes alone [4].

Different treatment methods have been used to remove dyes from wastewater. These include coagulation–flocculation, membrane filtration, advanced oxidation, photocatalysis, biodegradation, ion exchange, and adsorption [5]. Among these options, adsorption is widely used for dye removal because it is simple to operate and can achieve high removal efficiency. It also offers the possibility of regenerating and reusing the adsorbent [6]. Adsorption performance is strongly influenced by the properties of the adsorbent. Important factors include surface area, pore structure, particle size, surface functional groups, surface charge, and chemical stability [7]. For this reason, developing reusable adsorbents from waste-derived or naturally abundant materials is increasingly considered a useful strategy. Such materials can contribute to more sustainable wastewater treatment.

Plastic waste has also become a serious global sustainability issue, with polyethylene terephthalate (PET) forming an important part of this waste stream [8]. PET is widely used in bottles, packaging, fibres, films, and engineering products. Its broad use is related to its good mechanical strength, chemical resistance, transparency, and ease of processing [9]. PET is durable and does not degrade easily under natural conditions. As a result, it can persist for long periods in both terrestrial and aquatic environments. Global plastic production increased from 234 million tonnes in 2000 to 460 million tonnes in 2019. During the same period, plastic waste rose from 156 million tonnes to 353 million tonnes, while only about 9% was ultimately recycled after accounting for recycling losses [10]. If current trends continue, global plastic waste is expected to nearly triple by 2060, with less than one-fifth projected to be recycled [11]. These figures underline the need to convert PET waste into value-added materials instead of relying mainly on conventional recycling and disposal routes.

Recycled PET (rPET) can also be used as a starting material for adsorbent preparation. This provides a way to reuse PET waste while addressing dye contamination in water [3]. PET contains ester groups and aromatic rings that may interact with aromatic dye molecules through polar, hydrophobic, and possible π–π interactions [12]. Previous studies have shown that waste PET can be converted into porous adsorbents and composite materials for dye removal. For example, a recycled PET/sodium alginate composite prepared by a phase-separation method showed good MB adsorption and cycling performance [13]. Its adsorption behaviour was associated with electrostatic and polar interactions. These results support the use of PET-derived materials in wastewater treatment. However, PET alone may provide relatively few accessible adsorption sites and has limited surface polarity. Its adsorption performance can therefore be improved by modification or by combining it with an inorganic or naturally occurring adsorbent phase [14].

Sepiolite is a naturally occurring fibrous magnesium silicate clay with a high specific surface area and porous structure [15]. Its surface also contains silanol and hydroxyl groups, while the material shows good chemical and thermal stability. The tunnel-like structure of sepiolite makes it suitable for adsorption and ion-exchange applications, including interactions with organic pollutants [16]. It has been studied for the removal of both cationic and anionic dyes from water, and its adsorption behaviour is often evaluated using Langmuir and Freundlich isotherm models [3]. However, pristine sepiolite can agglomerate and may be difficult to handle or recover after adsorption. Incorporating it into a polymer matrix such as rPET may improve structural stability and processability while retaining the adsorption-related properties of the clay [3]. In a composite, rPET acts as the polymer framework, while sepiolite contributes surface hydroxyl groups, porosity, and potential sites for dye adsorption.

Combining rPET with sepiolite offers a way to link PET waste valorisation with the adsorption properties of the clay [3]. In this study, post-industrial rPET was dissolved in a trifluoroacetic acid/dichloromethane (TFA/DCM) solvent system and then blended with sepiolite using a solution-blending method. Interactions at the polymer–clay interface may influence composite formation and the accessibility of adsorption sites. These interactions, however, should be assessed through physicochemical characterisation rather than inferred only from the preparation method.

PET-based and sepiolite-based adsorbents have mostly been studied as separate systems. Only limited work has combined post-industrial rPET with sepiolite for MB removal while also optimising the composite formulation and adsorption conditions. Some PET-derived adsorbents require additional functional polymers, energy-intensive processing, or chemical modification. In contrast, many sepiolite-based studies mainly focus on modifying the clay without addressing plastic-waste valorisation [17]. Few studies have combined preliminary one-factor-at-a-time screening with statistical optimisation using response surface methodology [3]. Response surface methodology, particularly the Box–Behnken design, can assess the individual, interaction, and quadratic effects of several variables with fewer experiments than a full-factorial design [3]. This is useful for adsorption studies because contact time, dye concentration, adsorbent dosage, pH, and temperature can influence one another in a nonlinear manner.

Recent adsorbent development has increasingly focused on highly engineered surface architectures that provide target-specific binding and rapid separation. For example, Yang et al. developed a core–shell magnetic ion-imprinted material using thiol–ene click polymerisation for selective ReO_4_^−^ capture. The material combined a magnetic Fe_3_O_4_-based core, an imprinted functional layer, and N-vinylimidazole-derived recognition sites, with adsorption attributed to synergistic electrostatic and hydrophobic recognition within specifically engineered cavities [18]. In contrast, the present rPET/sepiolite material is not designed as a target-specific imprinted adsorbent. Instead, it combines a waste-derived rPET framework with naturally occurring sepiolite, providing a heterogeneous polymer–clay surface through a comparatively simple solution-blending route. The distinction of the present approach therefore lies in integrating post-industrial PET valorisation with clay-based adsorption functionality and subsequent multivariable process optimisation, rather than relying on magnetic separation, template imprinting, or highly specific recognition chemistry. Because the target pollutants and adsorption mechanisms differ, direct comparison of adsorption capacities between these systems is not appropriate; nevertheless, the comparison illustrates the different design strategies available for developing functional adsorbents for aqueous pollutant removal.

Based on our preliminary work [3], the present study develops and systematically evaluates an rPET/sepiolite composite adsorbent for MB removal from aqueous solution. The composite formulation was first optimised by examining rPET weight percentage, TFA solvent ratio, sepiolite loading, and blending time. The selected formulation was subsequently characterised using particle size analysis, surface area and porosity measurements, scanning electron microscopy, and Fourier-transform infrared spectroscopy. Adsorption performance was then statistically optimised using response surface methodology based on a Box–Behnken design by varying contact time, initial MB concentration, adsorbent dosage, pH, and temperature. Finally, reusability, adsorption kinetics, equilibrium isotherms, thermodynamic behaviour, and possible adsorption mechanisms were evaluated. The principal contribution of this work is the integration of post-industrial PET valorisation with natural-clay functionality through a solution-blended composite, together with systematic formulation screening, multivariable process optimisation, reusability assessment, and adsorption modelling for MB removal.

## 2. Results and Discussion

### 2.1. Characterisation of rPET/Sepiolite Adsorbent

#### 2.1.1. Textural Analysis

Particle size analysis was carried out for rPET, sepiolite, and the rPET/sepiolite composite. Table 1 summarises the particle size distribution of each sample based on the measured intensity percentage.

Table 1 shows that rPET had an average particle size of 926.97 nm. Sepiolite showed a larger particle size of 1067.45 nm, with an intensity of 87.4%. In comparison, the rPET/sepiolite composite had a much smaller average particle size of 267.10 nm. The decrease in particle size is consistent with reduced aggregate size and better dispersion of the composite components. This may increase the accessible surface area available for adsorption. Smaller particles may also shorten the diffusion path and thereby improve the rate of dye uptake. Similar effects of particle size reduction on adsorption performance have been reported in previous studies [19]. For example, Mosthofa et al. reported that reducing the particle size of activated carbon increased its surface area and pore volume. This change was accompanied by a higher adsorption capacity for MB. The improvement was mainly related to the greater external surface area available at smaller particle sizes [20].

As shown in Table 2, the rPET/sepiolite composite had a BET specific surface area of 0.67 m^2^/g and a total pore volume of 0.003925 cm^3^/g. For neat rPET, the corresponding values were 0.35 m^2^/g and 0.000826 cm^3^/g. The composite also showed an average pore size of 26.05 nm. Its surface area was higher than that of neat rPET but remained much lower than that of pristine sepiolite (295.11 m^2^/g). This suggests that incorporation of sepiolite into the rPET matrix reduced the amount of clay surface accessible to nitrogen during BET analysis. The pronounced decrease relative to pristine sepiolite suggests that a substantial fraction of the clay surface became embedded, covered, or otherwise inaccessible after incorporation into the rPET matrix. Consequently, the adsorption behaviour of the composite should not be interpreted as involving the full internal surface area of pristine sepiolite; accessible external and interfacial sites are likely to play an important role.

#### 2.1.2. Proposed Interaction Mechanism of rPET/Sepiolite Adsorbent

**Figure 1:**
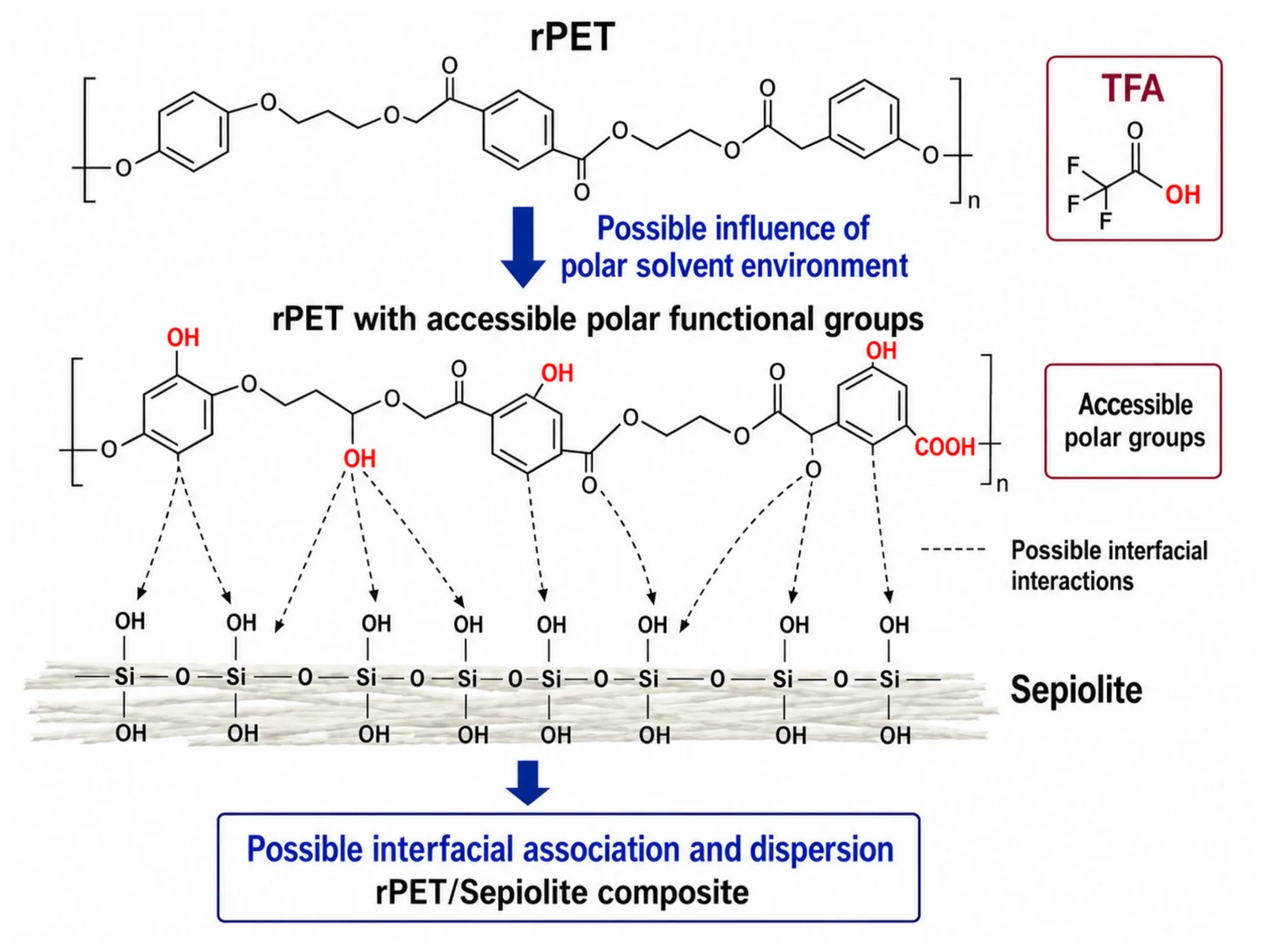
Conceptual schematic of possible interfacial interactions between rPET and sepiolite based on the available functional group information.

Figure 1 illustrates the proposed interaction between rPET and sepiolite. During dissolution of rPET in the TFA/DCM system, the polar solvent environment may alter the accessibility of ester and terminal hydroxyl/carboxyl groups at the polymer surface. These polar groups may participate in interfacial interactions with sepiolite [3,16]. Sepiolite contains surface silanol groups (Si–OH), which may contribute to hydrogen bonding and other interfacial interactions with polar functional groups of rPET [16]. Similar polar interfacial interactions have been discussed for sepiolite-containing polymer systems, although their extent depends on polymer chemistry, clay dispersion, and processing conditions [16]. Such interactions may contribute to interfacial compatibility and dispersion within the composite. Accordingly, the interactions illustrated in Figure 1 are presented as plausible interfacial associations based on the available functional group information and should not be interpreted as direct spectroscopic confirmation of PET hydrolysis or specific chemical bond formation.

#### 2.1.3. Morphological Analysis

**Figure 2:**
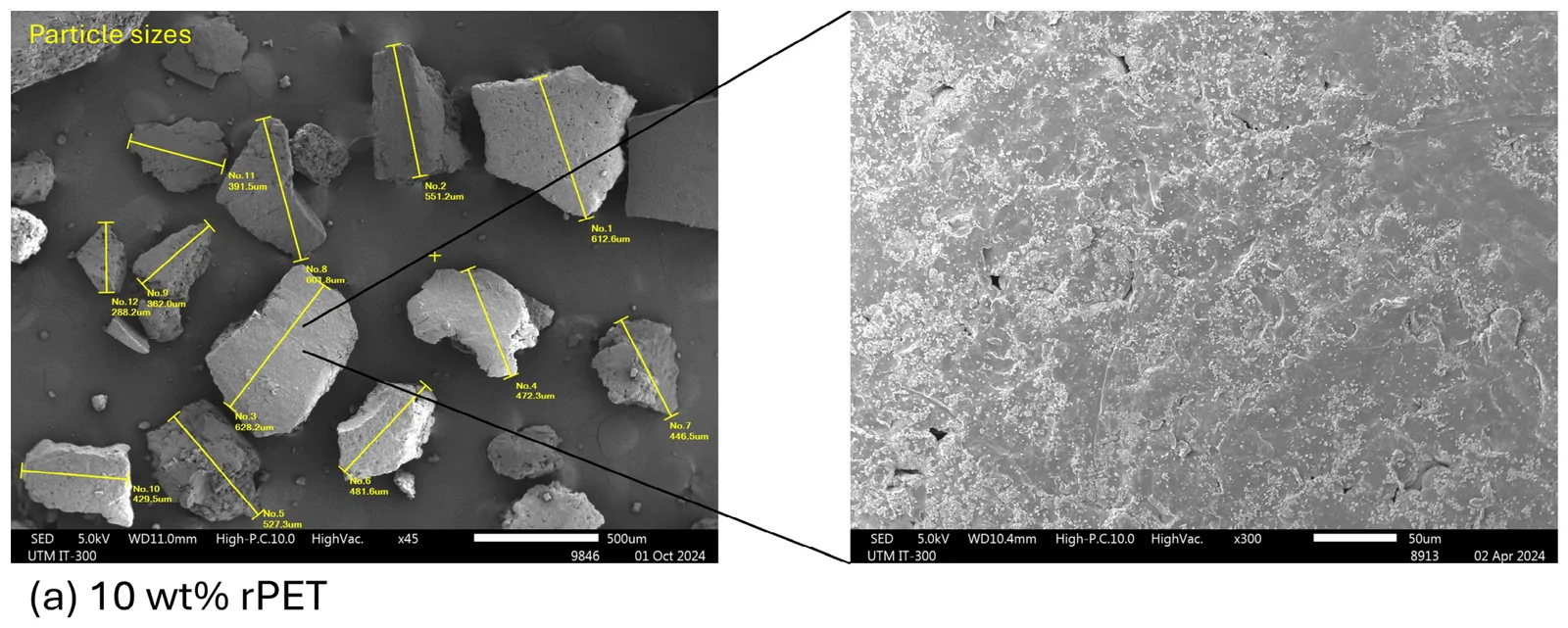

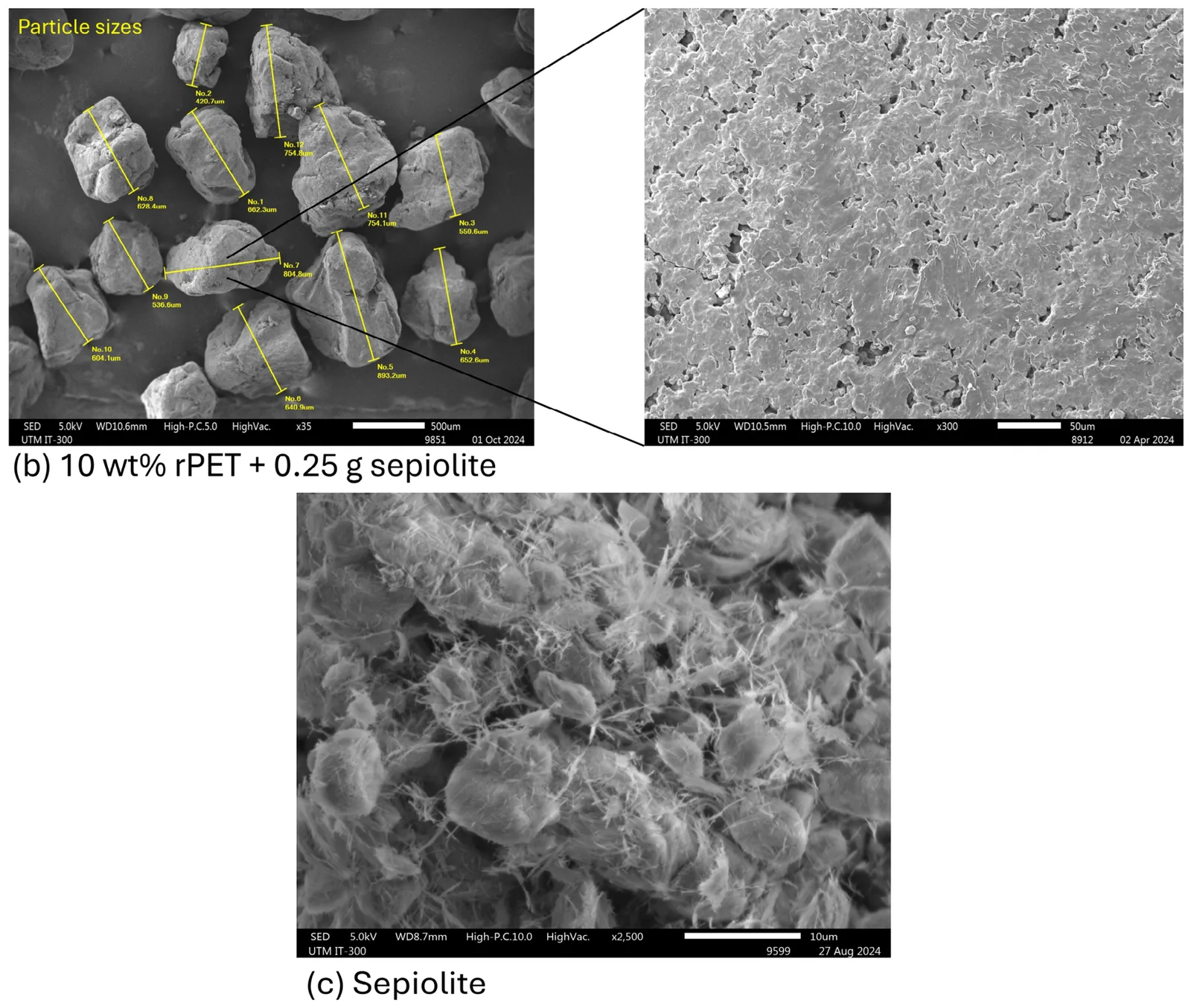
Surface morphology of (**a**) 10 wt% rPET without sepiolite, (**b**) rPET/sepiolite composite prepared using 10 wt% rPET and 0.25 g sepiolite, and (**c**) sepiolite powder.

The addition of a non-solvent reduces the solvent proportion, causing thermodynamic instability and phase separation at the polymer interface, forming a polymer-rich matrix and a polymer-poor pore region [21]. SEM images in Figure 2 show that variation in the TFA:DCM ratio significantly influenced the surface porosity and morphology of the PET matrix [22]. Tsai and Liu (2021) noted that DCM’s volatility leads to skin formation, which resists solvent and nonsolvent diffusion, slowing lamination and precipitation, thus reducing macro-voids [23]. Surface morphology varies with different solvent compositions, affecting porosity and texture. Figure 2a shows the surface morphology of 10 wt% rPET prepared without sepiolite. The neat rPET exhibited an irregular porous morphology with interconnected macrovoids of approximately 4.7–13.1 µm and rough polymer regions. In comparison, Figure 2b shows the rPET/sepiolite composite prepared using 10 wt% rPET and 0.25 g sepiolite. The composite retained a porous morphology, with interconnected voids of approximately 2.7–17.1 µm, while fine granular features associated with the incorporated clay were observed within the polymer matrix. These observations indicate that incorporation of sepiolite modified the surface texture while preserving the porous structure of the rPET matrix.

Meanwhile, Figure 2c shows a SEM image of sepiolite in powder form. The material exhibits a characteristic fibrous morphology, which is typical of naturally occurring sepiolite. The image shows numerous needle-like fibrils intertwined and forming loosely connected bundles. These fibres appear to grow from and cluster around plate-like particulate cores, creating a highly entangled, three-dimensional network. The high-aspect-ratio fibres are consistent with the characteristic needle-like morphology of sepiolite reported in the literature [15,16]. The open and loosely packed arrangement provides abundant inter-fibre voids, which are beneficial for adsorption processes because they facilitate dye penetration and access to internal adsorption sites. The open fibrous arrangement provides inter-fibre voids that may facilitate solution penetration and access to adsorption sites.

The irregular fibre agglomerates suggest that sepiolite retained its characteristic morphology without obvious collapse or fragmentation [9]. This structural integrity is important because the fibrous surface can provide access to silanol (Si–OH) groups involved in dye adsorption. The SEM images therefore show that the sepiolite maintained its expected microfibrous and loosely aggregated structure. These features support its use as an adsorptive and reinforcing phase in the rPET/sepiolite composite.

#### 2.1.4. Functional Group Analysis

**Figure 3:**
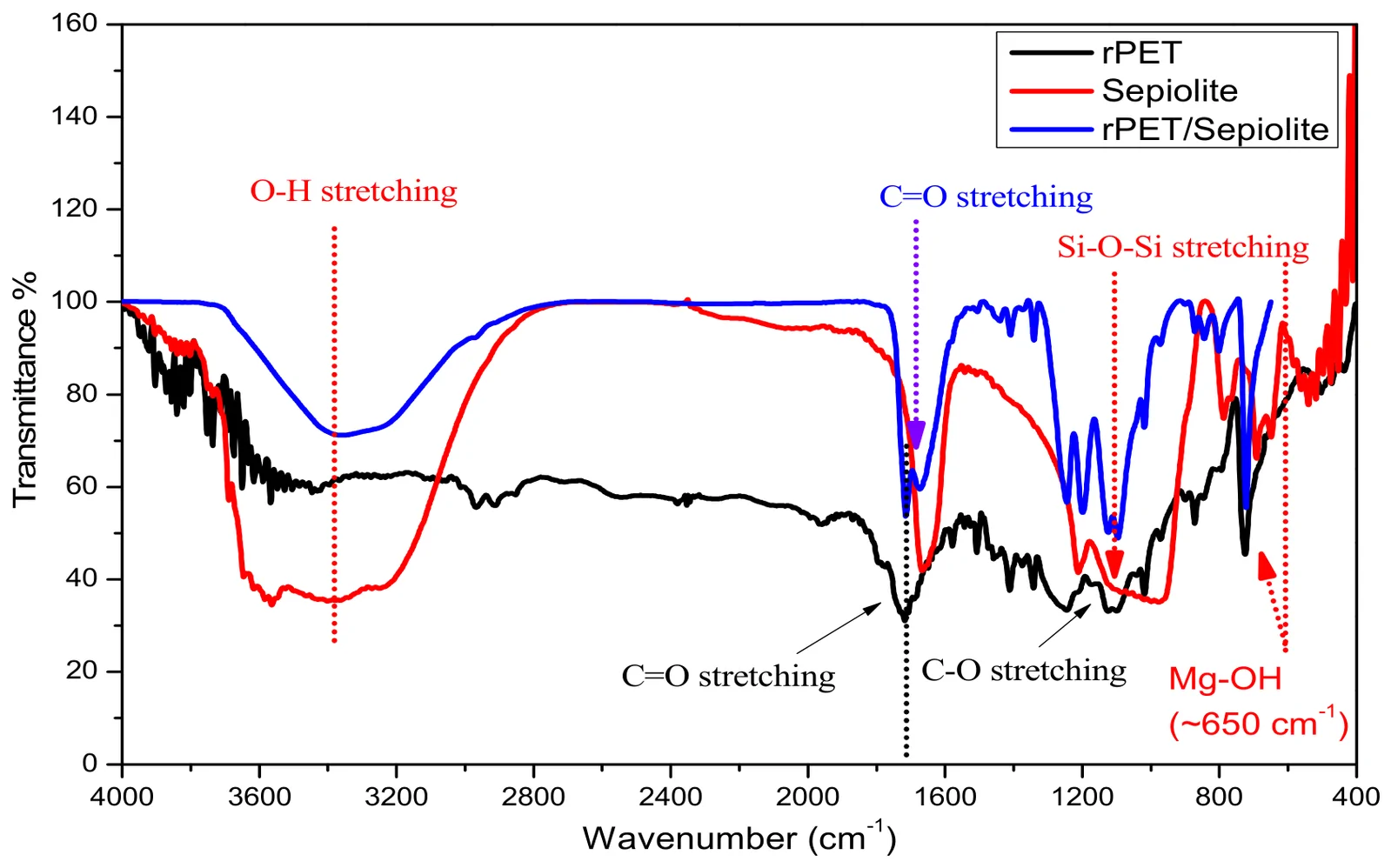
IR spectra of rPET, sepiolite, and rPET/sepiolite adsorbent.

Figure 3 presents the FTIR spectra of rPET, sepiolite, and the rPET/sepiolite composite over the range of 4000–500 cm^−1^. The rPET spectrum showed the characteristic ester-related bands, including strong carbonyl (C=O) and C–O stretching vibrations, consistent with the characteristic functional groups of PET-based materials [3]. In contrast, the sepiolite spectrum was mainly characterised by silicate-related bands, particularly Si–O–Si vibrations, together with O–H- and Mg–OH-related bands characteristic of the clay structure [16,24]. The composite spectrum contained features associated with both the rPET and sepiolite components, supporting their coexistence in the prepared material [3]. Overall, the FTIR results confirm the presence of polyester- and silicate-related functional groups in the rPET/sepiolite composite, rather than demonstrating formation of new chemical bonds.

### 2.2. RSM Optimisation of MB Adsorption

The BBD was employed to evaluate the effects of five variables on MB removal by the rPET/sepiolite adsorbent: contact time (A, 5–120 min), MB concentration (B, 1–50 mg/L), adsorbent amount (C, 0.1–5 g), pH (D, 2–12), and temperature (E, 30–80 °C). The complete 46-run experimental design, together with the actual and predicted MB removal values, is provided in Table S1.

Equation (1) was then applied to establish an empirical relationship between the independent variables and their corresponding response values based on the experimental results.

Adsorption (%) = +99.03 + 2.02 A − 5.77 B + 33.55 C + 4.46 D + 1.78 E + 2.75 AB +0.1975 AC − 2.75 AD − 1.94 AE + 9.94 BC + 4.09 BD + 3.24 BE − 13.49 CD − 5.30 CE +2.35 DE − 3.79 A^2^ − 2.83 B^2^ − 35.03 C^2^ + 0.2621 D^2^ − 2.49 E^2^ (1)

The fitted equation was used to describe MB removal within the investigated factor ranges, subject to the model-adequacy considerations discussed in Section 2.2.1. Among the linear terms, adsorbent dosage (C) showed the strongest positive contribution, whereas MB concentration (B) showed a negative effect on percentage removal. The response also showed important interaction effects, particularly BC and CD, while the large negative quadratic coefficient for C^2^ indicated pronounced curvature in the adsorbent-dosage response and the presence of an optimum within the investigated range [25,26,27]. Detailed statistical significance of the individual, interaction, and quadratic terms is discussed in Section 2.2.1 and provided in Table S2.

**Figure 4:**
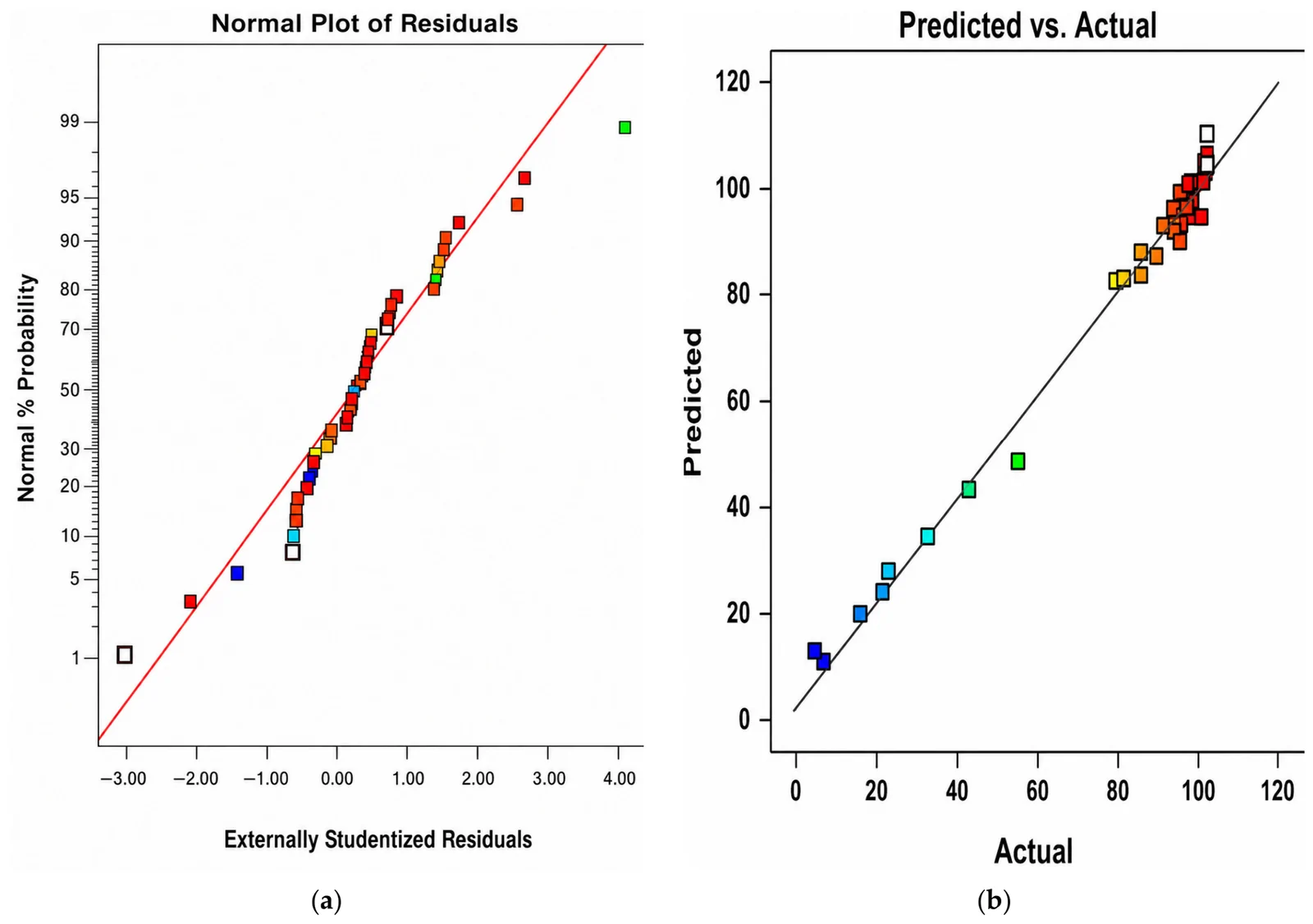
Model diagnostic plots for MB removal by the rPET/sepiolite adsorbent: (**a**) normal probability plot of residuals and (**b**) predicted versus actual responses.

Figure 4 presents the model diagnostic plots. The studentised residuals generally followed the expected linear trend in the normal probability plot (Figure 4a), while the predicted responses were generally close to the experimental values (Figure 4b). However, this visual agreement should be interpreted together with the significant lack-of-fit identified by ANOVA, as discussed in Section 2.2.1 [28].

#### 2.2.1. Analysis of Variance and Model Adequacy

Table S2 summarises the ANOVA results for the five-factor model, which included contact time (A), MB concentration (B), adsorbent dosage (C), pH (D), and temperature (E). The overall model was statistically significant (F = 114.18, *p* < 0.0001). The R^2^ value of 0.9892 indicates that 98.92% of the variation in MB removal was explained by the fitted model. The adjusted R^2^ (0.9805) and predicted R^2^ (0.9568) were also reasonably close. In addition, the adequate precision value of 37.25 indicated a strong signal relative to experimental noise.

Among the individual factors, MB concentration (B), adsorbent dosage (C), and pH (D) were highly significant (*p* < 0.0001). Contact time (A) was also significant (*p* = 0.0417), whereas temperature (E) was not significant at the 5% level (*p* = 0.0709). Several interaction terms, including BC (*p* < 0.0001), BD (*p* = 0.0396), CD (*p* < 0.0001), and CE (*p* = 0.0095), were statistically significant at the 5% level. The strongest effects were observed for the interaction between MB concentration and adsorbent dosage (BC, *p* < 0.0001) and between adsorbent dosage and pH (CD, *p* < 0.0001).

Among the interaction terms, AB (*p* = 0.1568), AC (*p* = 0.9174), AD (*p* = 0.1572), AE (*p* = 0.3140), BE (*p* = 0.0981), and DE (*p* = 0.2247) were not statistically significant at the 5% level. In contrast, BD remained significant (*p* = 0.0396). Among the quadratic terms, A^2^ (*p* = 0.0065), B^2^ (*p* = 0.0362), and C^2^ (*p* < 0.0001) were significant, whereas D^2^ (*p* = 0.8390) and E^2^ (*p* = 0.0625) were not significant. The particularly strong C^2^ term indicates pronounced curvature in the effect of adsorbent dosage on MB removal.

Following consideration of model reduction, the hierarchical quadratic model was retained for the present analysis. A backwards-elimination assessment of the existing dataset showed that removal of the nonsignificant interaction and quadratic terms did not improve overall model adequacy; the adjusted R^2^ decreased from 0.9805 to approximately 0.9768, while the lack-of-fit remained significant. The lack-of-fit test for the reported quadratic model gave F = 90.90 (*p* < 0.0001), indicating that some systematic variation across the full experimental design space was not completely described by the fitted model. Accordingly, the high R^2^ statistics should be interpreted together with the significant lack of fit, and model predictions are considered within the investigated factor ranges. Independent experimental validation of the selected operating condition is discussed in Section 2.2.3.

#### 2.2.2. Response Surface Analysis

**Figure 5:**
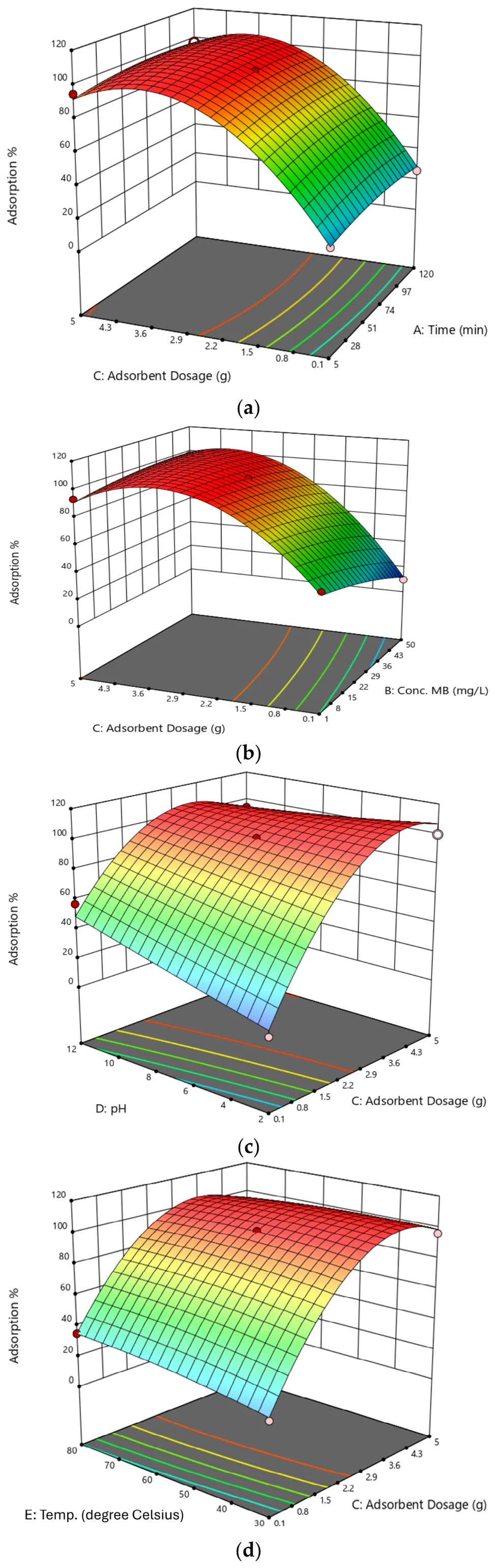
Three-dimensional response surface plots showing the combined effects of (**a**) contact time and adsorbent dosage (AC), (**b**) MB concentration and adsorbent dosage (BC), (**c**) pH and adsorbent dosage (CD), and (**d**) temperature and adsorbent dosage (CE) on MB removal efficiency.

Figure 5 presents the 3D response surfaces, showing how selected process variables affected MB removal by the rPET/sepiolite adsorbent. Figure 5a shows the combined effects of contact time (A) and adsorbent dosage (C). Increasing the adsorbent dosage produced a marked increase in MB removal, whereas the effect of contact time was smaller. Both factors showed significant individual effects, but their interaction (AC) was not statistically significant (*p* = 0.9174). The increase in removal with adsorbent dosage can be related to the larger number of available adsorption sites. A similar dosage-dependent trend has been reported for Cd^2+^ adsorption using modified red mud, where increasing adsorbent dosage improved removal efficiency because of the greater availability of adsorption sites [29]. This example is included only to support the general influence of adsorbent dosage on adsorption site availability and is not intended as a direct comparison with the present MB/rPET–sepiolite system.

Figure 5b shows the combined effect of MB concentration (B) and adsorbent dosage (C) on removal efficiency. At higher MB concentrations, the percentage removal tended to decrease when the adsorbent dosage was low. Increasing the dosage reduced this effect and improved MB removal. This trend can be explained by the greater number of adsorption sites available when more adsorbent is present. At higher dye concentrations, the available sites may become insufficient if the adsorbent dose is limited. Increasing the adsorbent dosage provides more sites for MB uptake and can therefore maintain a higher removal efficiency. Similar behaviour has been reported for MB adsorption using rice husk, where increasing adsorbent dosage increased dye removal because more surface area and adsorption sites became available [30].

Figure 5c shows the combined effect of adsorbent dosage (C) and pH (D) on MB removal. Increasing the adsorbent dosage generally improved removal across the investigated pH range. However, the magnitude of this improvement varied with pH, indicating that the two factors did not act independently. This behaviour can be related to changes in both the number of available adsorption sites and the surface charge of the adsorbent. A similar trend was reported for phenol removal using a geotextile/activated carbon (GTX/AC) adsorbent. In that study, increasing the adsorbent dosage up to 16 g produced smaller changes in removal efficiency at both pH 2 and pH 12. This suggests that the effect of adsorbent dosage can depend on pH because pH influences the ionisation of the adsorbate and the surface charge of the adsorbent [31].

Figure 5d shows the combined effect of adsorbent dosage (C) and temperature (E) on MB removal. Temperature was not significant as an individual factor. However, the interaction between dosage and temperature (CE) was significant (*p* = 0.0095). Increasing the adsorbent dosage generally improved MB removal across the investigated temperature range. This can be related to the greater number of adsorption sites available at higher dosages. The response surface also suggests that the effect of dosage varied with temperature. Similar observations have been reported in previous adsorption studies, where an optimum adsorbent dosage was identified despite changes in temperature [29].

#### 2.2.3. Numerical Optimisation of Adsorption Conditions

**Figure 6:**
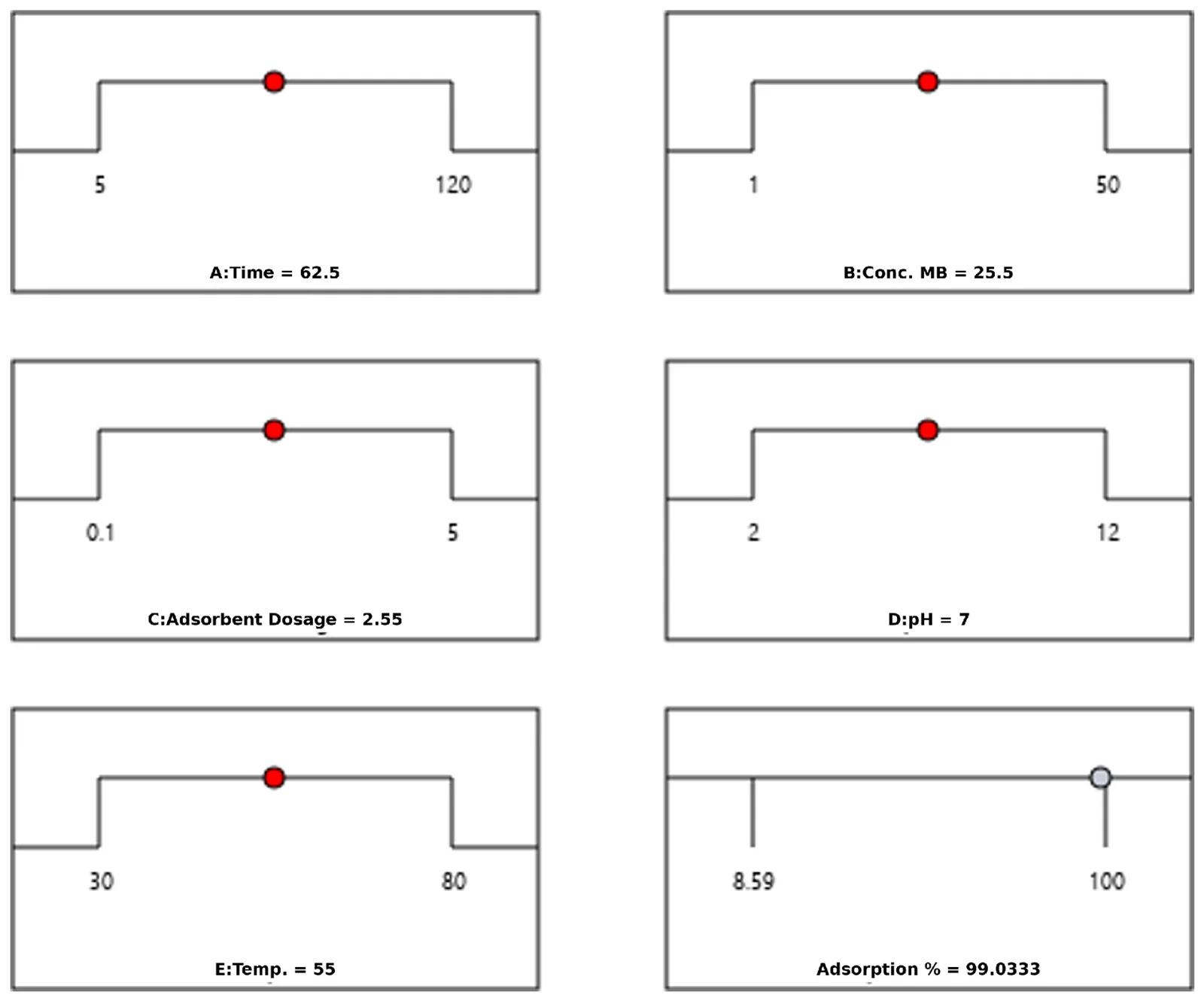
Numerical optimisation of operating conditions for MB removal using the rPET/sepiolite adsorbent.

Figure 6 summarises the effect of the five BBD factors on MB removal by the rPET/sepiolite adsorbent. These factors were contact time (A), MB concentration (B), adsorbent dosage (C), pH (D), and temperature (E). An independent experimental validation was performed at the selected operating conditions of 62.5 min contact time, 25.5 mg/L MB concentration, 2.55 g adsorbent in 50 mL solution, pH 7, and 55 °C. The experimentally measured MB removal efficiency was 99.06%, compared with the model-predicted value of 99.03%. This corresponds to an absolute deviation of approximately 0.03 percentage points and a relative deviation of approximately 0.03%, demonstrating close agreement between the predicted and experimental responses at the selected operating condition. The BBD also included six centre-point runs at the same nominal operating condition, which gave MB removal efficiencies of 99.3, 99.2, 99.0, 99.2, 99.4, and 98.2%, corresponding to a mean of approximately 99.05 ± 0.44% (SD). These centre-point runs provide an indication of experimental repeatability at the selected condition, while the independently recorded validation experiment gave 99.06% removal compared with the model prediction of 99.03%.

Across the 46 BBD runs, MB removal ranged from 8.6% to 100%. The lowest removal of 8.6% occurred at pH 2 with an adsorbent dosage of 0.1 g. This result shows that both pH and adsorbent dosage affected the adsorption response. The response surface further indicated that increasing the adsorbent dosage could partly offset the lower removal observed under less favourable pH conditions within the studied range. The wide variation in removal across the experimental runs supports the use of RSM to identify suitable operating conditions for MB adsorption [27].

### 2.3. Reusability Performance

**Figure 7:**
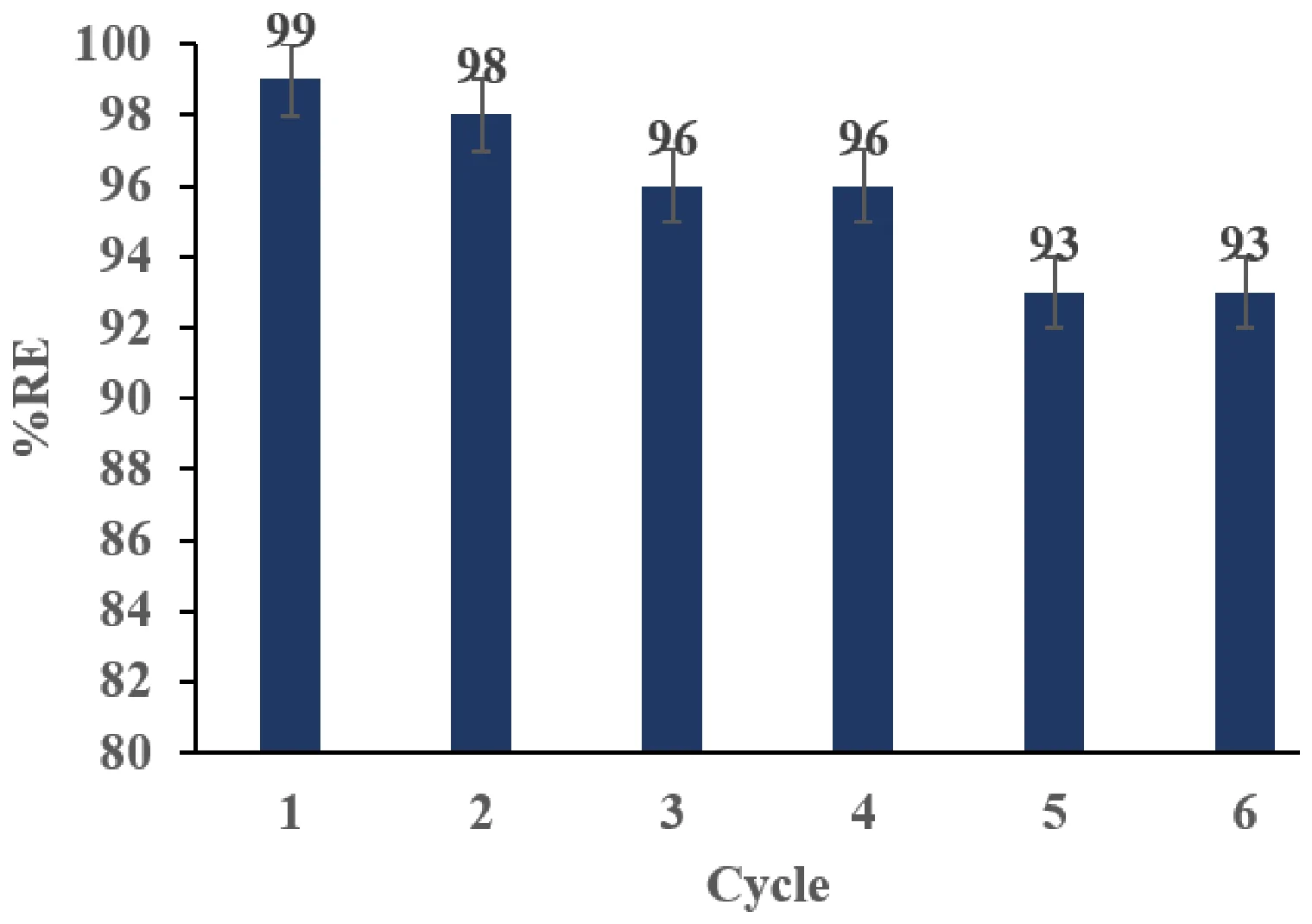
Reusability efficiency of the rPET/sepiolite adsorbent over six consecutive MB adsorption–washing–reuse cycles.

Figure 7 shows the reusability of the rPET/sepiolite adsorbent over six consecutive adsorption–washing–reuse cycles. Reusability is important because it indicates whether the adsorbent can maintain its performance after repeated use [32]. After each adsorption cycle, the rPET/sepiolite adsorbent was washed several times with distilled water and subsequently reused with fresh MB solution. Reusability efficiency (%RE) was calculated after each cycle using Equation (8). The adsorbent maintained more than 90% reusability efficiency throughout all six cycles. The reusability efficiency decreased gradually from 99% in Cycle 1 to 93% in Cycle 6, with values of 98%, 96%, 96%, 93%, and 93% recorded for Cycles 2–6, respectively [33]. The reusability efficiency decreased gradually from 99% in Cycle 1 to 93% in Cycle 6. Despite this decline, the adsorbent maintained a reusability efficiency above 90% throughout all six cycles [33]. Because the concentration of MB released into the washing solution was not quantified, the distilled-water washing step cannot be considered evidence of complete or quantitative desorption. Accordingly, the reported %RE values represent retention of adsorption performance after washing and reuse rather than desorption efficiency. Physical carryover or incomplete removal of previously adsorbed MB cannot be excluded.

Although the rPET/sepiolite adsorbent retained more than 90% reusability efficiency over six adsorption–washing–reuse cycles, this result demonstrates short-term retention of adsorption performance rather than direct preservation of the material structure. Post-cycle SEM, FTIR, and BET analyses were not performed in the present study; therefore, changes in surface morphology, functional groups, or pore characteristics after repeated reuse cannot be directly assessed. Future studies should combine extended reuse testing with post-cycle morphological, chemical, and textural characterisation to evaluate the structural stability of the composite during repeated operation. The gradual decrease in %RE cannot be assigned to a specific mechanism from the available data. Incomplete removal of retained MB during distilled-water washing and physical loss of adsorbent during repeated handling may be possible contributing factors; however, neither MB desorption nor adsorbent mass recovery was quantitatively measured in the present study [34,35,36]. Small losses of adsorbent during repeated handling, filtration, and washing may also contribute to the gradual decline in reusability performance. Future work should quantify the MB released during the washing/desorption step to determine desorption efficiency and evaluate more effective regeneration/desorption protocols.

### 2.4. Adsorption Modelling

Adsorption modelling was used to examine the kinetic, equilibrium, and thermodynamic behaviour of MB on the rPET/sepiolite adsorbent. The kinetic analysis included the pseudo-first-order (PFO), pseudo-second-order (PSO), and intraparticle diffusion models. Equilibrium data were evaluated using the Langmuir and Freundlich isotherm models. Thermodynamic parameters were also calculated to assess the spontaneity and thermal nature of the adsorption process.

#### 2.4.1. Adsorption Kinetics

Kinetic experiments were carried out to follow the rate of MB adsorption as equilibrium was approached. The experimental data were fitted using three models: pseudo-first-order (PFO), pseudo-second-order (PSO), and intraparticle diffusion, as given in Equations (9)–(11) [37]. The fitted kinetic parameters are summarised in Table 3.

**Figure 8:**
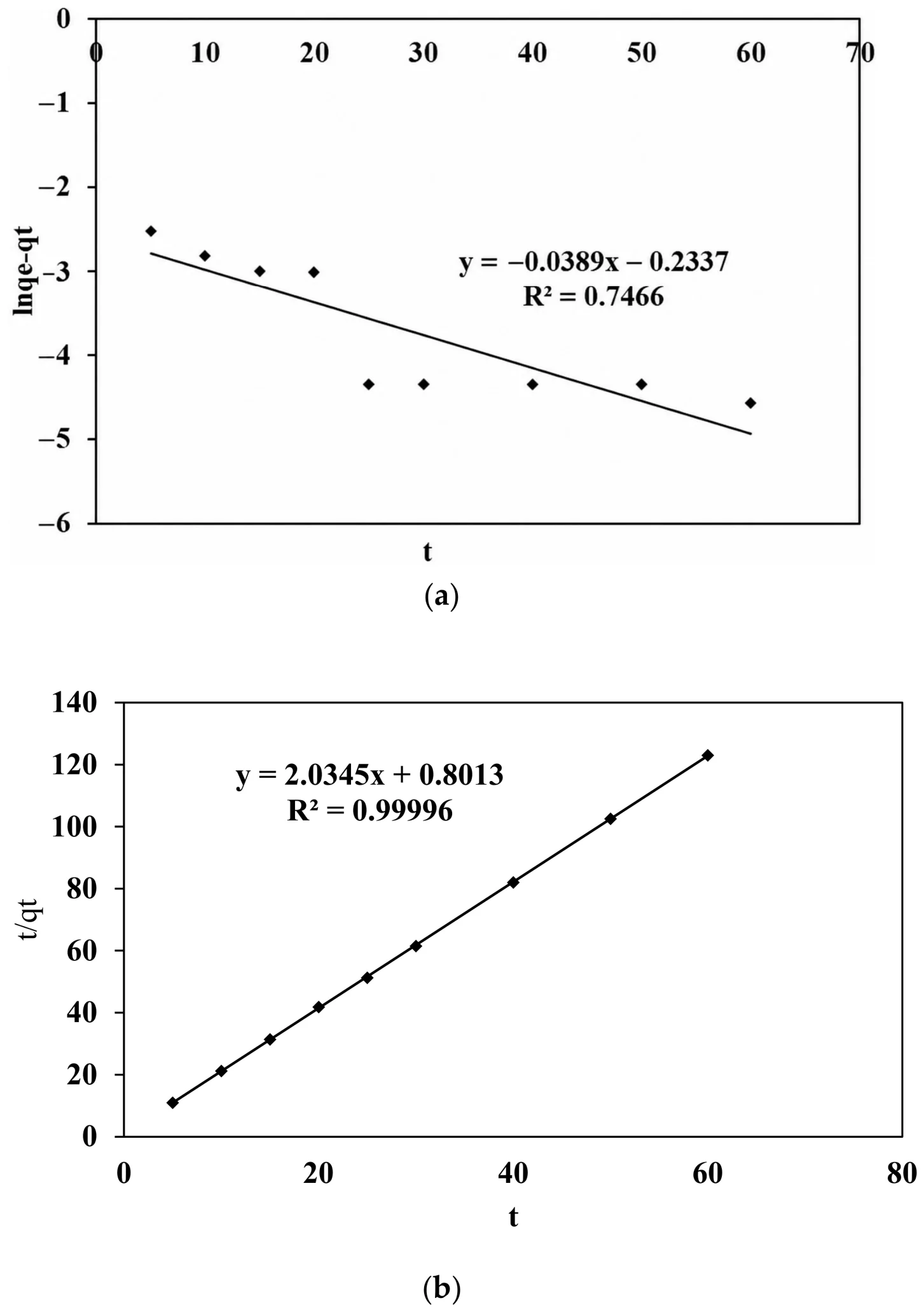

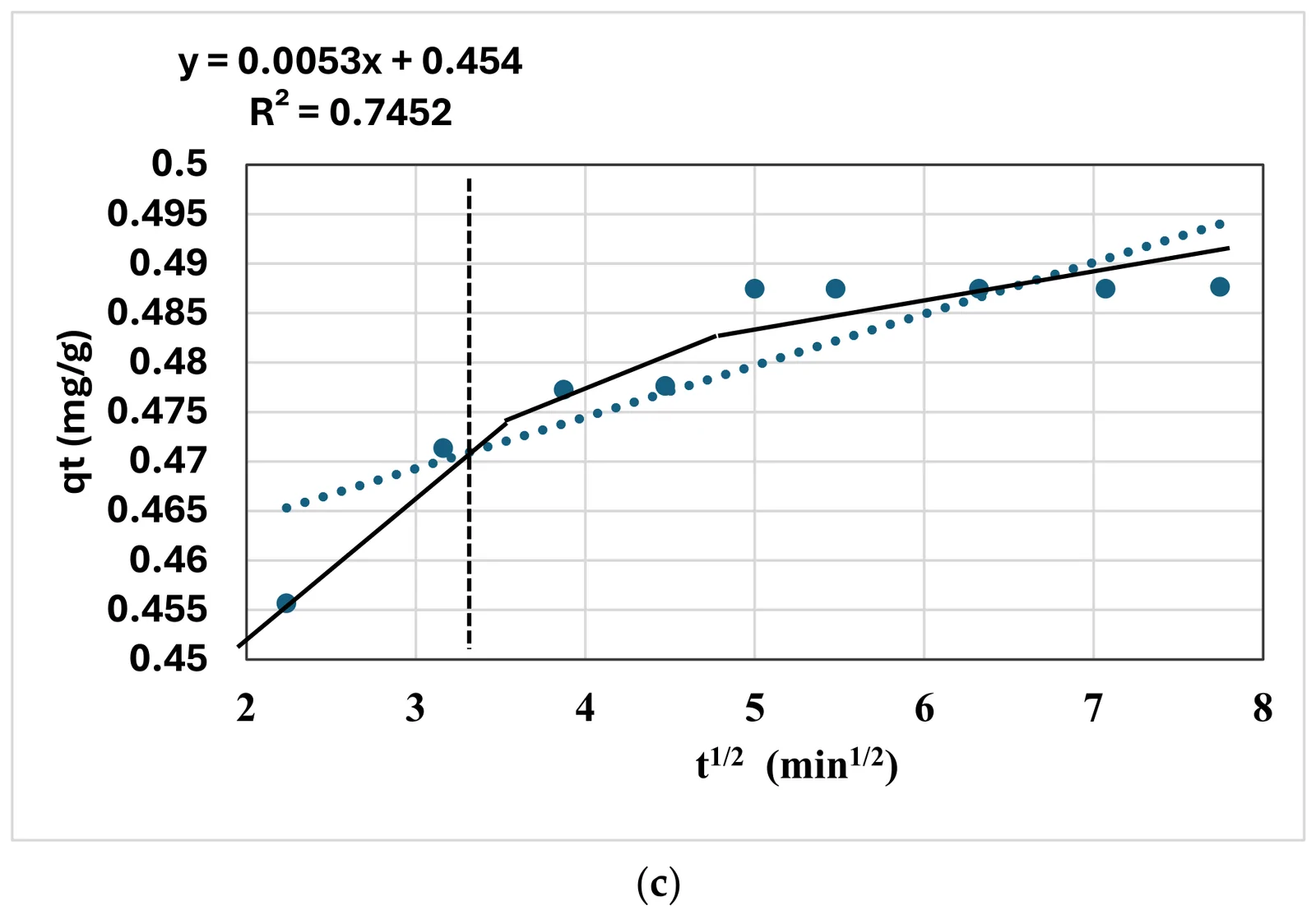
Linearised kinetic plots for MB adsorption onto the rPET/sepiolite adsorbent: (**a**) pseudo-first-order, (**b**) pseudo-second-order, and (**c**) Weber–Morris intraparticle diffusion model.

The PFO and PSO kinetic fits are presented in Figure 8a,b, respectively. The PSO model showed a much closer agreement with the experimental data, with an R^2^ value of 0.99996 compared with 0.7466 for the PFO model. The experimental equilibrium adsorption capacity was approximately 0.488 mg/g, while the PSO model gave a calculated equilibrium adsorption capacity of approximately 0.492 mg/g, showing close agreement between the experimental and calculated values. This agreement suggests that the PSO model describes the adsorption rate well under the studied conditions. The good fit may be related to the availability and accessibility of adsorption sites on the rPET/sepiolite surface [33,38]. However, fitting the PSO model alone is not sufficient to conclude that chemisorption is the only mechanism involved, since other mass-transfer and surface-interaction processes may also contribute to MB uptake.

To further examine mass-transfer behaviour, the Weber–Morris intraparticle diffusion model was applied by plotting *q_t_* against *t*^0.5^. If intraparticle diffusion were the only rate-controlling step, the plot would be expected to pass through the origin. However, Figure 8c did not pass through the origin and deviated from a single linear relationship. The non-zero intercept of 0.454 mg/g suggests that boundary-layer effects also contributed to MB uptake. The relatively moderate R^2^ value of 0.7452 further indicates that intraparticle diffusion alone cannot fully describe the adsorption process. MB uptake was relatively rapid at the beginning and then became slower as equilibrium was approached. The faster initial uptake may be related to the greater availability of accessible surface sites, while the later decrease in adsorption rate can occur as these sites become occupied and diffusion into less accessible regions becomes more important. Therefore, the kinetic behaviour suggests that MB adsorption involved more than one mass-transfer step, including external surface adsorption and intraparticle diffusion, as also reported for other adsorption systems [39,40,41].

#### 2.4.2. Isotherm Study

Adsorption isotherms were used to describe how MB was distributed between the solution and the adsorbent at equilibrium. They also provide information about adsorption capacity and the nature of the adsorbent surface. The Langmuir model assumes adsorption on a uniform surface with equivalent sites and formation of a monolayer. In contrast, the Freundlich model is empirical and is commonly used for heterogeneous surfaces containing sites with different adsorption affinities [42].

To evaluate the equilibrium behaviour of MB adsorption, the experimental data were fitted to the linearised Langmuir and Freundlich models. The quality of each fit was assessed using the coefficient of determination (R^2^), and the calculated parameters are listed in Table 4. Within the present linearised comparison, the Freundlich model showed greater consistency with the experimental equilibrium data, as reflected by its higher R^2^ value; however, this comparison should be regarded as descriptive rather than as definitive model discrimination. This behaviour is consistent with the mixed nature of the rPET/sepiolite composite, where the polymer and clay phases provide chemically and structurally different adsorption environments [43]. The Freundlich model is expressed as

(2) $$q_{e}=K_{F}\times C_{e}^{1/n}$$

where *q_e_* is the amount of MB adsorbed per unit mass of adsorbent at equilibrium, *C_e_* is the equilibrium concentration of MB in solution, *K_F_* is the Freundlich constant associated with adsorption capacity, and *n* is the heterogeneity factor.

**Figure 9:**
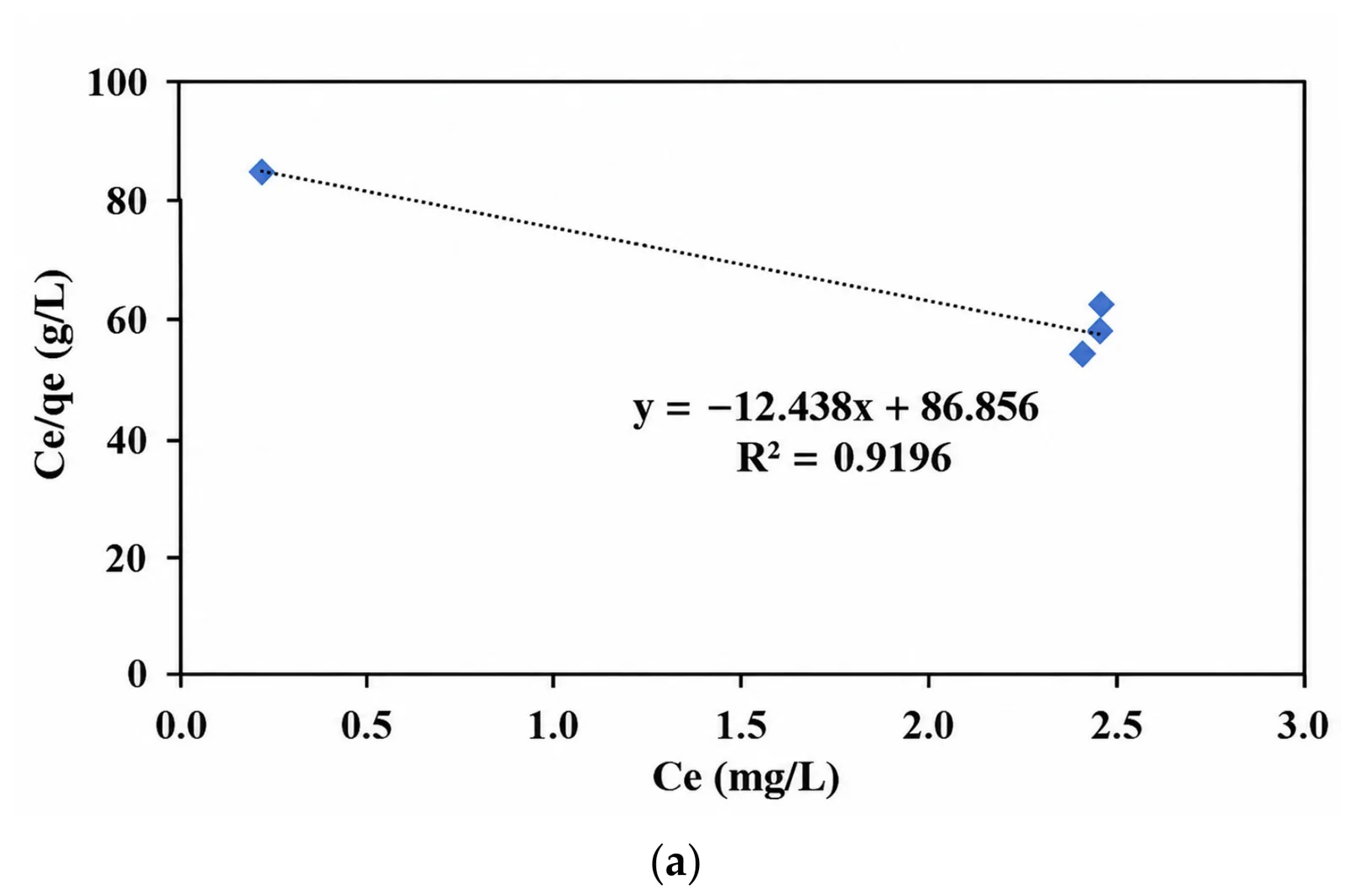

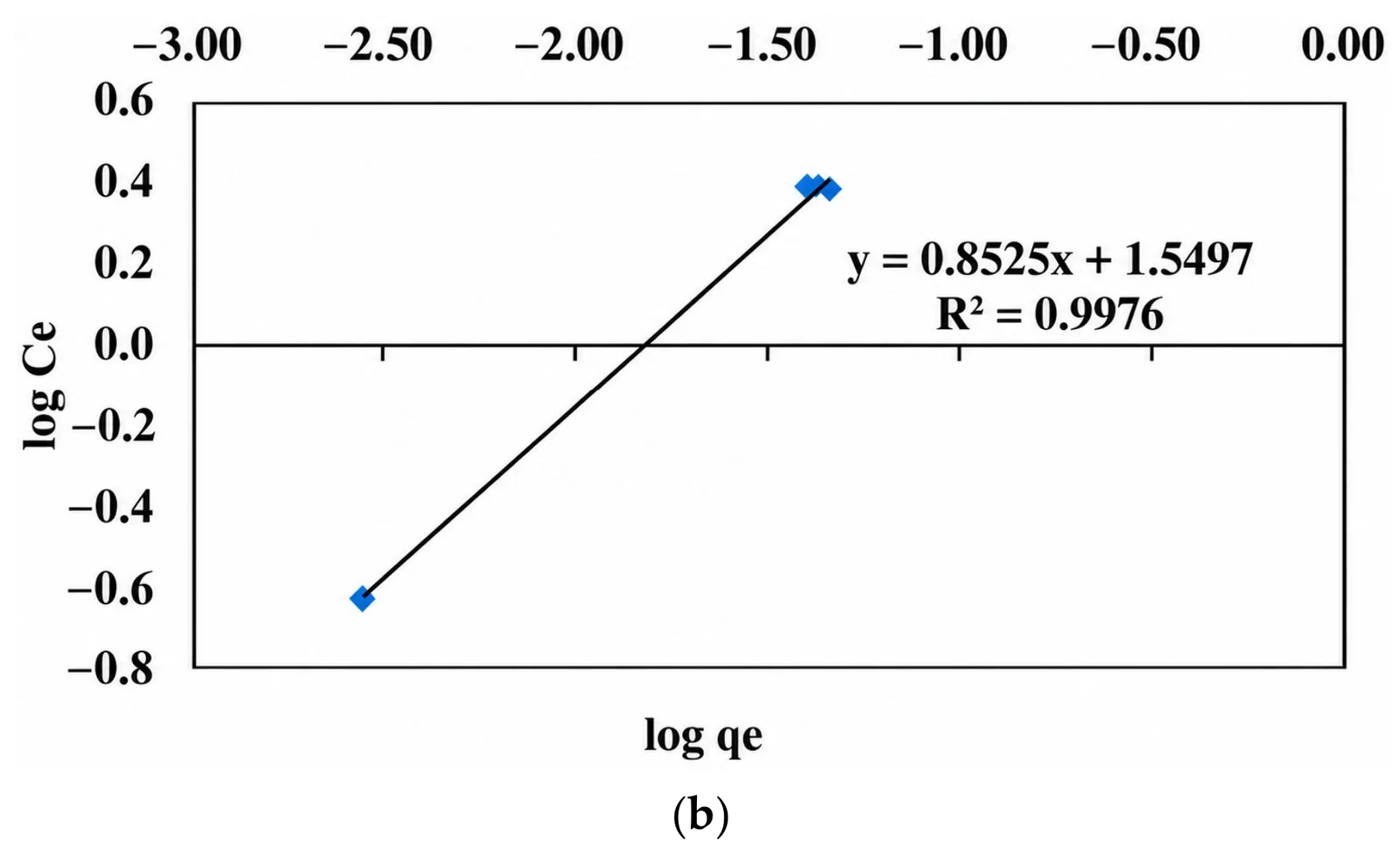
Linearised isotherm plots for MB adsorption onto the rPET/sepiolite adsorbent: (**a**) Langmuir and (**b**) Freundlich models.

In Figure 9, for the Freundlich model, the data were represented using the inverse linearised form, *loglog C_e_* = *nloglog q_e_* − *nloglog K_F_*. Based on the coefficients of determination, the Freundlich model provided a better fit (*R*^2^ = 0.9976) than the Langmuir model (*R*^2^ = 0.9196). The higher linearised *R*^2^ value for the Freundlich model suggests that the equilibrium data are more consistent with a heterogeneous-site description within the present analysis, although this result should not be interpreted as conclusive evidence of a unique adsorption mechanism. In the Freundlich framework, adsorption sites may possess different affinities, with higher-affinity sites being preferentially occupied as the adsorbate concentration increases [43]. Although the Langmuir model produced an *R*^2^ of 0.9196, the negative slope of the linearised *C_e_*/*q_e_* versus *C_e_* plot resulted in non-physical Langmuir parameter estimates, indicating that the model was not suitable for quantitative interpretation under the investigated conditions. Similar Freundlich behaviour has been reported for MB adsorption using heterogeneous adsorbent materials [44]. A more rigorous comparison should include direct nonlinear fitting of the equilibrium data together with complementary error functions such as RMSE and *χ*^2^, and this is recommended for future investigation.

#### 2.4.3. Thermodynamic Study

Thermodynamic parameters were used to evaluate the favourability and thermal nature of MB adsorption onto the rPET/sepiolite adsorbent. Following verification of the equilibrium-constant definition and unit treatment, the thermodynamic parameters were recalculated using the dimensionless equilibrium ratio described in Section 3.7.3. The calculated ΔG° values were −8.93, −9.23, and −9.24 kJ/mol at 303.15, 328.15, and 353.15 K, respectively as shown in Table 5. Within the adopted thermodynamic framework, the negative ΔG° values indicate that MB adsorption was thermodynamically favourable over the investigated temperature range [45,46]. The calculated ΔH° value of −6.99 kJ/mol indicates that the adsorption process was exothermic [45]. The positive ΔS° value of +0.00653 kJ/(mol·K) indicates a small increase in disorder at the solid–liquid interface during adsorption. Although the ΔG° values became slightly more negative across the investigated temperature range, this small change should not be interpreted as evidence that increasing temperature produced a statistically significant increase in MB removal. In the RSM analysis, temperature (E) was not significant as an individual factor (*p* = 0.0709), although the interaction between adsorbent dosage and temperature (CE) was significant (*p* = 0.0095). The two analyses therefore describe different aspects of the system: the thermodynamic analysis indicates that adsorption remained favourable over the investigated temperature range, whereas the RSM evaluates the statistical contribution of temperature to removal efficiency in the presence of the other process variables. Thermodynamic behaviour is dependent on the adsorbent–adsorbate system and experimental conditions, and different thermodynamic responses have been reported for adsorption systems in the literature [45,46,47]. These thermodynamic parameters are therefore interpreted specifically within the investigated experimental conditions and the equilibrium-constant definition described in Section 3.7.3.

#### 2.4.4. Proposed Adsorption Mechanism

The adsorption results suggest that MB uptake by the rPET/sepiolite adsorbent involves more than one mass transfer and surface interaction process. The PSO model provided the best kinetic fit; however, as discussed in Section 2.4.1, agreement with the PSO model alone does not establish chemisorption as the dominant mechanism. The Weber–Morris analysis further indicated that intraparticle diffusion was not the sole rate-controlling process, suggesting contributions from external surface uptake and diffusion within accessible regions of the adsorbent.

The better fit of the Freundlich model compared with the Langmuir model indicates that MB adsorption is more consistent with a heterogeneous distribution of adsorption sites with different affinities. Similar Freundlich behaviour has been reported for heterogeneous carbonaceous, clay-based, and polymeric adsorbents [48,49,50]. The Freundlich fit should therefore be interpreted primarily as evidence of surface heterogeneity rather than definitive proof of multilayer adsorption.

Textural characterisation also supports the contribution of accessible surface and pore regions to MB uptake. The nitrogen adsorption–desorption isotherm of the rPET/sepiolite adsorbent exhibited Type II behaviour with an H3 hysteresis loop, consistent with a non-rigid pore network and slit-like or interparticle voids. Such textural features may facilitate adsorbate access and mass transfer within the composite. However, given the much lower BET surface area of the composite compared with pristine sepiolite, pore-related uptake should be interpreted as occurring only within accessible pore or interparticle regions rather than throughout the original sepiolite internal surface. Thus, external surface and exposed interfacial adsorption are expected to contribute substantially to MB uptake. However, the nitrogen physisorption behaviour alone does not establish the molecular mechanism of MB adsorption.

Surface chemistry and pore characteristics are important in controlling dye adsorption, although their individual contributions depend strongly on adsorbent composition and preparation route. For example, Zhang et al. reported that activation of pig manure-derived carbon using different chemical and physical activation routes produced marked differences in pore structure and MB adsorption behaviour, demonstrating the strong influence of physicochemical surface characteristics on adsorbent performance [51]. In the present rPET/sepiolite system, FTIR confirms the coexistence of polyester-related and silicate-related functional groups, while surface-charge measurements provide information on the pH-dependent electrostatic behaviour of the composite. However, because surface-sensitive techniques such as XPS were not performed, specific chemical binding sites or individual molecular interactions cannot be assigned directly. Therefore, the adsorption interactions discussed here should be regarded as plausible contributions supported by the combined physicochemical characterisation and adsorption modelling rather than as direct spectroscopic confirmation of a unique adsorption mechanism.

Mechanistic interpretation based only on kinetic or isotherm model fitting should be treated cautiously because such models describe adsorption behaviour but do not directly identify individual molecular interactions. Complementary physicochemical characterisation can therefore provide important supporting evidence. For example, Li et al. investigated Cr(VI) adsorption onto citric-acid-modified peanut shell using adsorption modelling together with FTIR, XRD, carboxyl-group analysis, and zeta-potential measurements to discuss the possible adsorption mechanism [52]. In the present rPET/sepiolite system, the kinetic and equilibrium models are therefore interpreted together with FTIR, textural characterisation, and surface-charge measurements. The available FTIR results confirm the presence of polar functional groups associated with the polyester and silicate components; however, they do not independently confirm a specific hydrogen-bonding interaction with MB. Similarly, electrostatic attraction is considered a plausible contribution on the basis of the measured pH-dependent surface-charge behaviour rather than as a uniquely proven adsorption pathway.

Furthermore, electrostatic interactions may also contribute to the adsorption of cationic MB. The surface charge of the adsorbent depends on solution pH relative to its isoelectric point (IEP). The rPET/sepiolite adsorbent exhibited an IEP in the acidic region, approximately pH 3.48. At pH values above the IEP, the adsorbent surface is expected to become increasingly negatively charged, which can favour electrostatic attraction toward cationic MB molecules [53]. Since the selected adsorption conditions were near pH 7, electrostatic attraction may therefore contribute to MB uptake.

**Figure 10:**
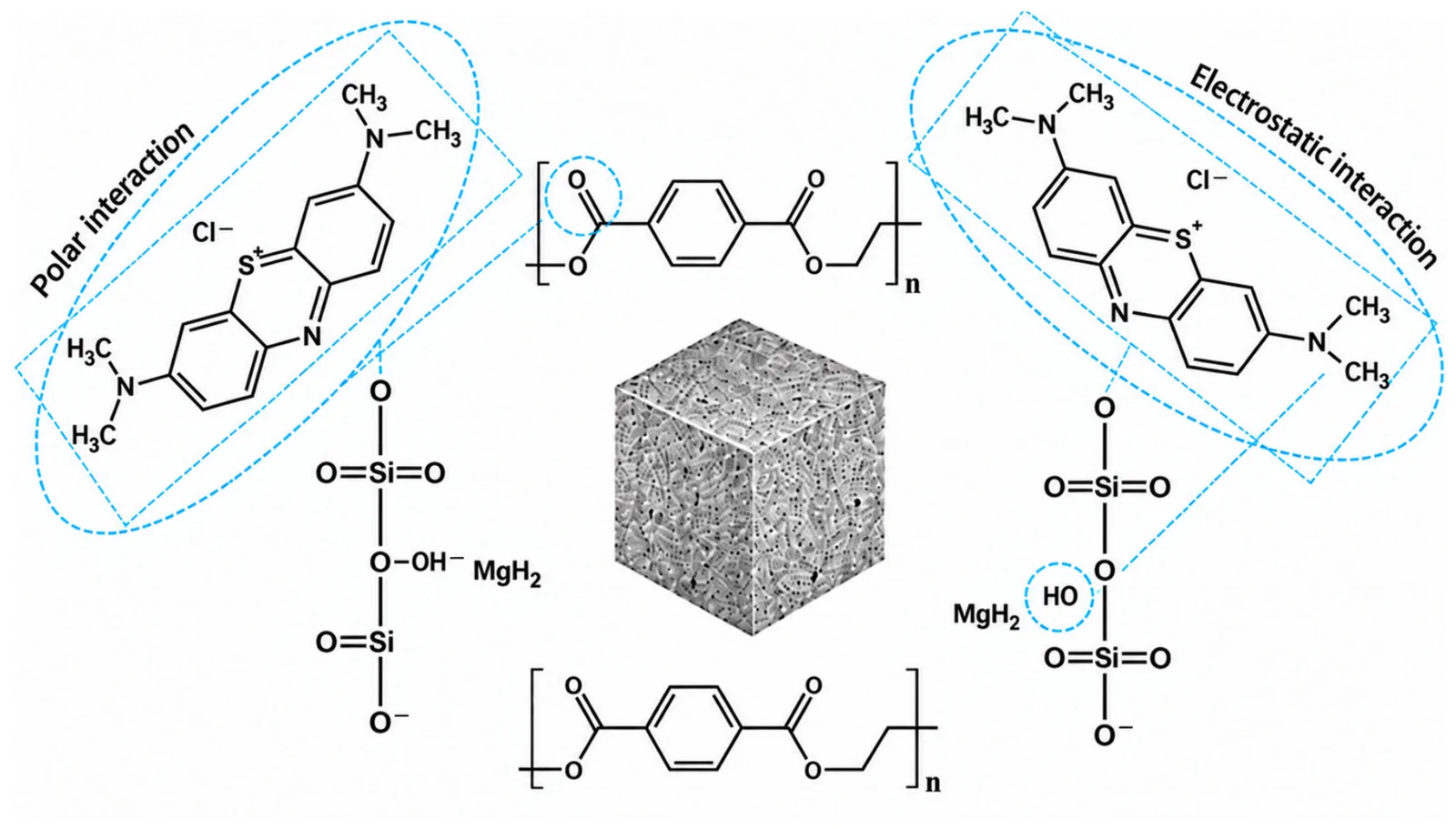
Schematic representation of the proposed and plausible adsorption interactions of MB with the rPET/sepiolite adsorbent based on the available characterisation and adsorption modelling.

A schematic representation of the proposed MB adsorption mechanism is presented in Figure 10. Based on the combined kinetic, isotherm, textural, and surface-charge observations, MB uptake is proposed to involve external mass transfer, diffusion into accessible regions of the composite, adsorption at heterogeneous surface sites, and electrostatic interactions [54]. These mechanisms are proposed based on the available characterisation and modelling results and should not be interpreted as direct proof of a single dominant adsorption pathway.

### 2.5. Comparative Performance Assessment

Table S3 compares the present rPET/sepiolite adsorbent with selected PET-derived and sepiolite-based materials previously investigated for dye adsorption; the corresponding literature sources are provided with Table S3 in the Supplementary Information. These studies employ different preparation routes, operating conditions, and adsorbent modifications, providing useful context for assessing the characteristics of the present composite. For example, PET/MWCNT composite membranes, PET waste-derived graphene, NiO-modified sepiolite, and ultrasonically treated sepiolite have all been investigated as dye adsorbents. These approaches demonstrate the versatility of PET-derived and sepiolite-based materials but differ substantially in synthesis requirements, material composition, and adsorption conditions.

In the present study, post-industrial rPET was combined with sepiolite through solution blending using a TFA/DCM solvent system, without the high-temperature pyrolysis required for some PET-derived carbon materials. A distinctive aspect of this work is the systematic evaluation of rPET weight percentage, solvent ratio, sepiolite content, and blending time during adsorbent formulation, followed by RSM-based optimisation of the adsorption operating conditions. The resulting composite exhibited a smaller average particle size and higher BET surface area and pore volume than neat rPET, while maintaining high MB removal and reusability under the investigated conditions. Kinetic and equilibrium modelling further provided information on the adsorption behaviour of the composite.

Although high MB removal efficiencies were obtained under the selected operating conditions, percentage removal alone does not provide a complete measure of adsorbent performance. Adsorption capacity is also influenced by the initial pollutant concentration, solution volume, and adsorbent dosage [7,49]. In the present study, the selected RSM condition used 2.55 g of adsorbent in 50 mL of 25.5 mg/L MB solution. At the model-predicted removal efficiency of 99.03%, these conditions correspond to a mass-balance adsorption capacity of approximately 0.495 mg/g; therefore, the high removal percentage should be interpreted in the context of the relatively high adsorbent-to-solution ratio. Accordingly, the adsorption capacity of the present composite is comparatively modest under the investigated conditions, and its main advantage lies in the valorisation of post-industrial PET through incorporation of a natural clay phase rather than exceptionally high adsorption capacity alone. Recent advanced adsorbents illustrate the importance of evaluating adsorption capacity together with surface properties and pollutant-specific interactions. For example, Xu et al. reported a Bi_2_MoO_6_ adsorbent for humic acid removal with a maximum adsorption capacity of approximately 63.1 mg/g, where the favourable performance was attributed to its specific surface area, positive surface charge, and abundant oxygen vacancies [55]. However, because humic acid and MB differ substantially in molecular structure, charge characteristics, and adsorption behaviour, and because the experimental conditions are different, direct numerical comparison of adsorption capacities is not appropriate. Such comparisons nevertheless demonstrate the importance of considering adsorption capacity, surface functionality, adsorbent dosage, and operating conditions collectively when assessing practical adsorbent performance.

Overall, the comparison indicates that the present rPET/sepiolite system provides a different route for combining post-industrial PET valorisation with clay-based adsorption. However, the environmental feasibility of the overall preparation route must also consider the use of the TFA/DCM solvent system during solution blending. The present study was conducted at laboratory scale, and solvent recovery or reuse was not evaluated experimentally; therefore, the current results should not be interpreted as demonstrating the environmental sustainability of the complete manufacturing process. For larger-scale implementation, recovery and reuse of the processing solvents would be important to reduce solvent consumption and associated waste generation. Additional considerations include solvent handling and containment, equipment compatibility, process safety, energy requirements for solvent removal, and the economic feasibility of solvent recovery. Direct numerical comparison among published adsorbents should also be interpreted cautiously because experimental conditions such as initial dye concentration, adsorbent dosage, contact time, pH, and temperature differ among studies [7,49]. Future work should therefore focus on standardised comparative testing; regeneration under realistic wastewater conditions; solvent recovery and reuse; reduction of solvent consumption; evaluation of alternative lower-impact processing routes, including partial hydrolysis and other chemical-recycling approaches; process economics; and scale-up feasibility.

## 3. Materials and Methods

### 3.1. Materials

Post-industrial rPET pellets were supplied by Alba Polyester Sdn. Bhd., Johor, Malaysia. The material had a molecular weight of 25,000 g/mol, a density of 1.38 g/cm^3^, and a melting point of 250 °C. Sepiolite was obtained as Pangel S9 from Tolsa, Madrid, Spain. Its reported properties included a molecular weight of 648 g/mol, density of 2.3 g/cm^3^, melting point of 1200 °C, surface area of 320 m^2^/g, moisture content of 6%, and viscosity of 3900 mPa·s at 25 °C. Trifluoroacetic acid (TFA), dichloromethane (DCM), sodium hydroxide (NaOH), and hydrochloric acid (HCl) were purchased from Sigma-Aldrich Chemistry, Selangor, Malaysia. Methylene blue (MB) was supplied by Hannschweiller Laboratory Chemicals, Germany. Distilled water was used for preparing all MB solutions.

### 3.2. One-Factor-at-a-Time (OFAT) Formulation Screening and Adsorbent Fabrication

The preparation of the rPET/sepiolite adsorbent was optimised using the one-factor-at-a-time (OFAT) approach. Table 6 summarises the formulation parameters evaluated for the rPET/sepiolite adsorbent. The initial ranges of rPET weight percentage, sepiolite content, and solvent ratio were selected based on the optimum conditions identified in our previous study [3].

**Figure 11:**
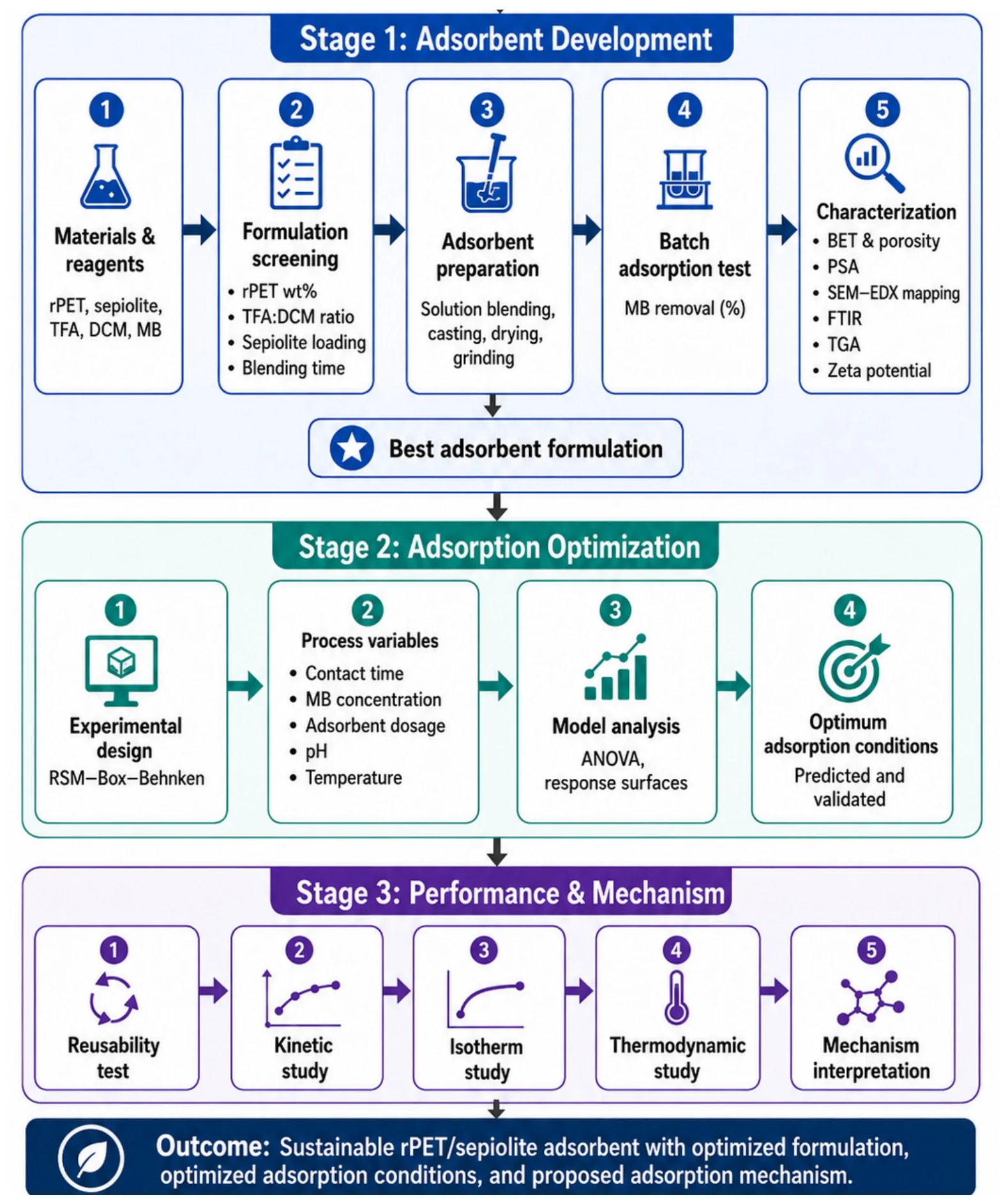
Overall flow chart and methodology for research.

Based on the OFAT screening, the selected formulation consisted of 10 wt% rPET, a TFA ratio of 3:7 (*v*/*v*), 0.25 g sepiolite, and a blending duration of 60 min. The resulting composite was cast, dried, ground, and sieved prior to adsorption experiments. Figure 11 displays the flow chart of the research methodology for the experiment, involving adsorbent preparation, optimisation of adsorption conditions using RSM, and determination of the adsorption mechanism. From the first stage of the experiment, the relationship between formulations and performances of rPET, sepiolite, and solvent was taken into consideration. Finally, the adsorption mechanism was evaluated using adsorption models, and the reusability of the adsorbent was assessed through repeated adsorption–washing–reuse cycles.

### 3.3. MB Adsorption Analysis for OFAT Screening

**Figure 12:**
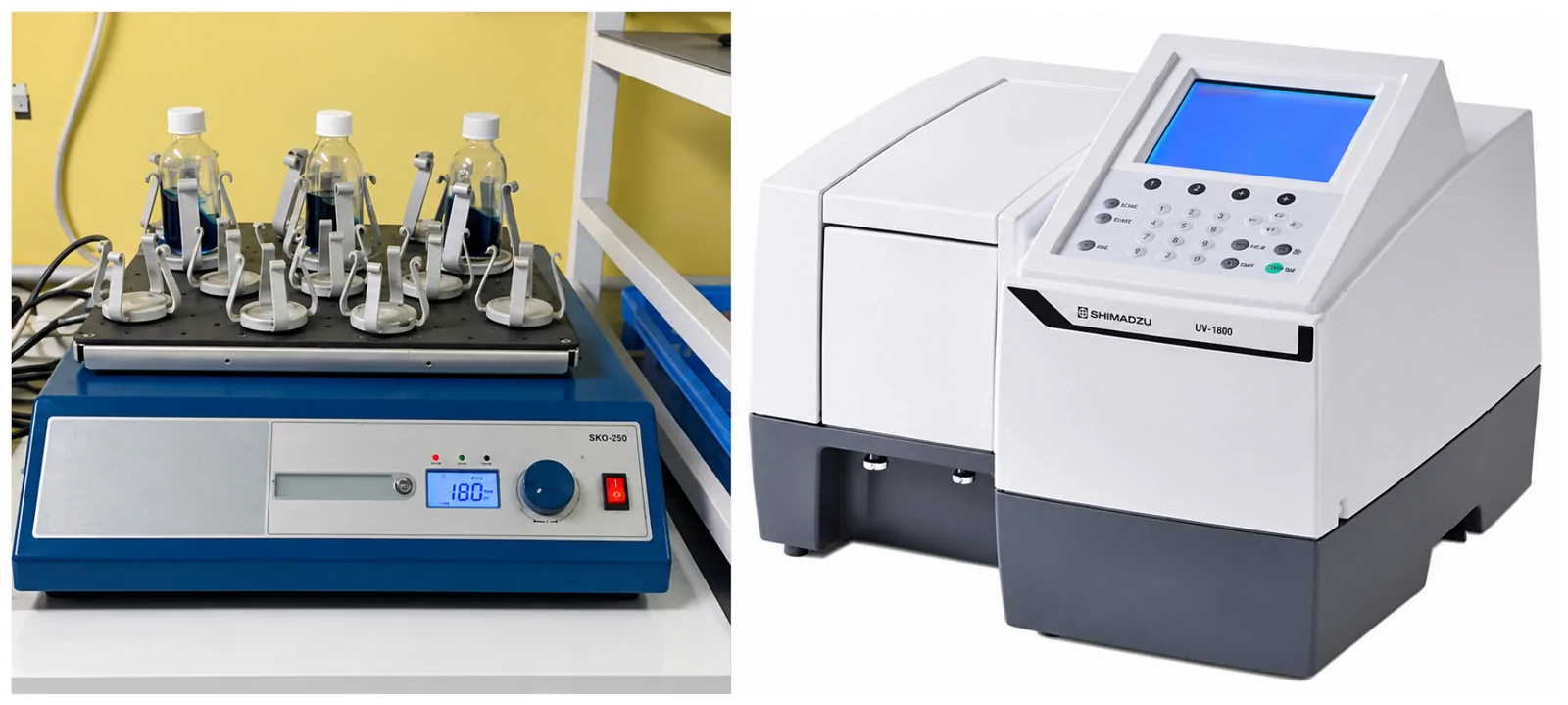
The orbital shaker and UV–vis spectrophotometer used in adsorption of MB dye.

A UV–vis spectrophotometer (Shimadzu UV-1800, Japan) was used to measure the MB concentration before and after adsorption, following our earlier procedure [3]. MB concentration was determined from absorbance measurements using a calibration curve based on the Beer–Lambert relationship. Figure 12 shows the orbital shaker and UV–vis spectrophotometer used in the adsorption experiments. For the formulation screening, 1.0 g of adsorbent was added to 50 mL of a 10 mg/L MB solution at pH 7. The samples were treated at room temperature for 60 min. After adsorption, the remaining MB concentration was measured at its maximum absorption wavelength (λmax) of 664 nm. The percentage of MB removed was then calculated using Equation (3):

(3) $$\frac{\mathrm{Ci}-\mathrm{Cf}}{\mathrm{Ci}}\times 100$$

where *C_i_* and *C_f_* are the initial and final MB concentrations, respectively, expressed in mg/L.

### 3.4. Characterisation of rPET/Sepiolite Adsorbent

The textural properties of the samples were evaluated using BET and Barrett–Joyner–Halenda (BJH) analyses, following the procedure reported previously [3]. These measurements were used to determine the specific surface area, average pore size, and pore size distribution. Surface morphology was examined using scanning electron microscopy (SEM). Functional groups were identified by Fourier-transform infrared spectroscopy (FTIR).

The surface area and total pore volume of the adsorbent were measured using BET and BJH adsorption methods, following our preliminary work [3]. BET analysis was carried out using a TriStar 2 surface area analyser (Micromeritics Instrument Corporation, Norcross, GA, USA). Nitrogen adsorption isotherms were recorded at 77 K. Before analysis, the samples were degassed at 130 °C under a vacuum (up to 10^−6^ Torr) for 12 h. The pore size distribution was obtained from the nitrogen adsorption data. Particle size distribution was measured using a Mastersizer 3000+ (Malvern Panalytical Ltd., Malvern, UK), which covers a particle size range of 0.01–3500 µm.

The surface morphology of the adsorbent was examined by scanning electron microscopy (SEM) using a Phenom ProX instrument (Phenom-World B.V., Eindhoven, The Netherlands). Images were obtained in secondary-electron mode using the upper and lower detectors. An accelerating voltage of 2 kV was applied to minimise possible surface damage. Before imaging, the samples were vacuum-dried and sputter-coated with a gold–palladium layer. Several regions of each sample were examined to assess the uniformity of particle size and pore structure. Energy-dispersive X-ray spectroscopy (EDX) and elemental mapping were reported in our earlier study [3] and used to identify the elemental composition and its distribution within the adsorbent.

Fourier-transform infrared (FTIR) spectroscopy was used to identify the functional groups present on the sample surface. Measurements were carried out using a Bruker ALPHA FTIR spectrometer (Bruker Optics GmbH & Co. KG, Ettlingen, Germany) over the wavenumber range of 500–4000 cm^−1^. For analysis, the powdered samples were mixed with KBr, which served as the supporting matrix.

Zeta potential measurements, as reported in our previous study [3], were used to examine the surface charge of the rPET/sepiolite adsorbent. Surface charge can influence the interaction between the adsorbent and cationic MB molecules. Changes in the magnitude and sign of the zeta potential can therefore provide information on possible electrostatic attraction or repulsion during adsorption. For the measurements, 0.1 g of adsorbent was mixed with 50 mL of a 50 mg/L MB solution and agitated at 150 rpm for 180 min. A separate sample containing 0.1 g of adsorbent in 50 mL of deionised water was agitated at 150 rpm for 24 h. The suspensions were allowed to settle for 5 min before measurement, following the procedure described by Batool et al. [56]. The measurement is based on the movement of charged particles under an applied electric field. Their electrophoretic mobility was related to the zeta potential using the Smoluchowski equation, given in Equation (4), and the more general Henry equation, given in Equation (5):

(4) $$\mu_{e}=\frac{\left(\varepsilon_{0}\varepsilon_{r}\zeta \right)}{\eta }$$

where *μ_e_* is the electrophoretic mobility, *ε*_0_ is the permittivity of vacuum, *ε_r_* is the relative permittivity (dielectric constant) of the medium, *ζ* is the zeta potential, and *η* is the dynamic viscosity of the medium.

(5) $$\mu_{e}=\frac{2\;\varepsilon_{0}\varepsilon_{r}\zeta \;f\left(\mathrm{\kappa a}\right)}{3\;\eta }$$

where *μ_e_* is the electrophoretic mobility, *ζ* is the zeta potential, *ε* is the dielectric permittivity, *η* is the viscosity, and *f*(*κa*) is Henry’s function describing the relationship between the particle radius *a* and the Debye length 1/*κ*. The zeta potential of the adsorbent was measured at 22 ± 1 °C using a Zeta Meter 4.0 (Zeta-Meter, Inc., Harrisonburg, VA, USA).

### 3.5. Batch Adsorption Experiments

During the 46 batch adsorption tests, the adsorbent was added to a bottle containing 50 mL of MB solution. The samples were maintained at the required experimental temperature and agitated at 180 rpm using a SHO-2D mechanical shaker (DAIHAN Scientific Co., Ltd., Wonju, Republic of Korea). The solution pH was adjusted to the required value using 0.1 mol/L HCl or 0.1 mol/L NaOH. A pH meter (Martini Instruments, Mi 150) was used to verify the pH. After adsorption, the suspension was filtered through 125 mm fast-grade filter paper. The residual MB concentration in the filtrate was measured using a UV–vis spectrophotometer following the procedure reported previously [3]. The percentage of MB removed was then calculated.

#### RSM-BBD Optimisation of Adsorption Conditions

Response surface methodology (RSM) was used together with a Box–Behnken design (BBD) to optimise MB adsorption by the rPET/sepiolite adsorbent. The experimental design was generated using Design-Expert software (version 13, Stat-Ease, Inc., Minneapolis, MN, USA). Each factor was studied at three levels, coded as −1, 0, and +1 for the low, centre, and high levels, respectively. Five independent variables were considered: adsorbent dosage, pH, temperature, initial MB concentration, and contact time. The objective was to identify the conditions that gave the highest MB removal efficiency. These factors were incorporated into the statistical model using the following equation:

(6) $$\mathrm{Xi}=\frac{x_{i}-x_{\mathrm{oi}}}{\Delta \mathrm{Xi}}$$

where *X_i_* is the coded value of the independent variable, *x_i_* is its actual experimental value, *x*_0*i*_ is the value at the centre point, and Δ*x_i_* represents the step change of that variable [57].

The adsorption behaviour of the rPET/sepiolite adsorbent was modelled and optimised using the RSM–BBD approach. The selected factor ranges were based on preliminary studies on adsorption optimisation [58,59]. The low, centre, and high levels were coded as −1, 0, and +1, respectively, as shown in Table 7. The response used for optimisation was the percentage removal of MB.

The experimental design consisted of 46 runs, including six centre-point replicates to estimate experimental variability. MB removal efficiency was calculated using Equation (3). The remaining MB concentration was measured by UV–vis spectrophotometry following the procedure reported previously [3]. RSM was then used to relate the response to the independent variables and their interaction effects. The experimental data were fitted to a second-order polynomial model, expressed as follows:

(7) $$Y=\beta_{0}\;+\;\sum_{i=1}^{k}\beta_{i}X_{i}\;+\;\sum_{i=1}^{k}\beta_{\mathrm{ii}}{X_{i}}^{2}\;+\sum_{i=1}^{k}\beta_{\mathrm{ij}}\;X_{i\;}X_{j}$$

In Equation (7), *Y* is the predicted response, *X_i_* and *X_j_* represent the coded independent variables, *k* is the number of independent variables, *β*_0_, is the intercept, *β_i_* represents the linear coefficients, *β_ii_* represents the quadratic coefficients, and *β_ij_* represents the interaction coefficients [60]. The magnitude and sign of these coefficients describe the contribution and direction of the corresponding model terms to the predicted response.

### 3.6. Reusability Study

For the reusability test, 2.55 g of rPET/sepiolite adsorbent was mixed with 50 mL of MB solution at an initial concentration of 25.5 mg/L and pH 7. The suspension was agitated at 55 °C for 62.5 min. After adsorption, the adsorbent was washed several times with distilled water to remove residual MB. The washed adsorbent was then shaken again for 62.5 min. It was subsequently dried in an oven and reused in the next adsorption cycle. The MB concentration in solution was measured after each cycle, and the reusability efficiency was calculated using the corresponding Equation (8):

(8) $$R_{n}=\frac{q_{n}}{q_{o}}\;\times \;100$$

The reusability efficiency of the adsorbent was evaluated over six adsorption–washing–reuse cycles. It was calculated by comparing the adsorption capacity after each reuse cycle (*q_n_*) with the initial adsorption capacity (*q*_0_). Both *q_n_* and *q*_0_ are expressed in mg/g. The procedure therefore evaluates retention of adsorption performance after distilled-water washing and reuse [61,62]. Because the concentration of MB released into the washing solution was not measured, quantitative desorption efficiency was not determined in the present study. Accordingly, reusability efficiency should not be interpreted as equivalent to desorption efficiency. The adsorbent mass was also not systematically recorded before and after each reuse cycle; therefore, quantitative mass recovery could not be determined.

### 3.7. Mathematical Models

The adsorption behaviour was further evaluated using kinetic, isotherm, and thermodynamic models. The kinetic analysis included the pseudo-first-order (PFO), pseudo-second-order (PSO), and intraparticle diffusion models to describe the adsorption rate and mass-transfer behaviour. Equilibrium data were analysed using the Langmuir and Freundlich isotherm models. Thermodynamic parameters were also evaluated to assess the favourability and spontaneity of the adsorption process.

#### 3.7.1. Kinetic Study

Adsorption kinetics were evaluated using three models: pseudo-first-order (PFO), pseudo-second-order (PSO) [63], and the intraparticle diffusion model [64]. These models were used to examine the adsorption rate and mass-transfer behaviour. The kinetic experiments were conducted under the selected adsorption conditions.

For each experiment, 2.55 g of rPET/sepiolite adsorbent was added to 50 mL of MB solution with an initial concentration of 25.5 mg/L. The suspension was maintained at 55 °C and agitated at 180 rpm. Samples were collected after 5, 10, 15, 20, 25, 30, 40, 50, and 60 min. The resulting data were analysed using the kinetic models listed in Table 8. The UV–vis concentration values used in the kinetic mass-balance calculations were corrected to the original batch-solution concentration basis before calculating adsorption capacity. Thus, *q_t_* and *q_e_* were calculated using an initial batch concentration of 25.5 mg/L, a solution volume of 0.050 L, and an adsorbent mass of 2.55 g.

The adsorption capacity of dye at equilibrium was determined by using Equation (12).

(12) $$q_{e}=[\frac{C_{i}-C_{e}}{M}]V$$

where *C_i_* and *C_e_* are the initial and equilibrium MB concentrations (mg/L), respectively. *V* is the solution volume (L) and *M* is the mass of adsorbent used (g). The amount of MB adsorbed per gram of adsorbent at time *t* was calculated using Equation (13):

(13) $$q_{t}=[\frac{C_{i}-C_{t}}{M}]V$$

where Ct is the concentration of dye at time t (mg/L).

#### 3.7.2. Isotherm Study

Equilibrium studies were conducted to evaluate MB adsorption onto the rPET/sepiolite adsorbent. The experiments were performed at a contact time of 62.5 min, adsorbent dosage of 2.55 g/50 mL, pH 7, and 55 °C, while the initial MB concentration was varied from 1 to 50 mg/L. Residual MB concentration was quantified using UV–vis spectrophotometry, and the equilibrium adsorption capacity was calculated using Equation (12). The resulting equilibrium data were then fitted to the Langmuir [65] and Freundlich [66] isotherm models using Equations (14) and (15), respectively, as presented in Table 9, while the present regression analysis was evaluated using their corresponding linearised forms as discussed in Section 2.4.2.

#### 3.7.3. Thermodynamic Study

Thermodynamic experiments were carried out at 30, 55, and 80 °C using the same general adsorption procedure [33]. The effect of temperature on MB adsorption was then evaluated. The thermodynamic parameters Gibbs free energy change (ΔG°), enthalpy change (ΔH°), and entropy change (ΔS°) were calculated using Equations (16)–(18). A plot of ln(K) against 1/T was used to obtain the required parameters, following the approach reported previously [63]. These values were used to assess the spontaneity of adsorption, the thermal nature of the process, and changes in disorder at the adsorbent–solution interface.

Δ*G*° = −*RT*ln*K_c_* (16)

(17) $$\ln K_{C}=\frac{-\Delta H{}^{\circ}}{\mathrm{RT}}+\frac{\Delta S{}^{\circ}}{R}$$

Δ*G*° = Δ*H*° − *T*Δ*S*° (18)

For the thermodynamic calculations, the equilibrium constant *K_c_* was treated as a dimensionless equilibrium ratio and was calculated as *K_c_* = (*C*_0_ − *C_e_*)/*C_e_*, where *C*_0_ and *C_e_* are the initial and equilibrium MB concentrations, respectively. Because *C*_0_ and *C_e_* were expressed in the same units (mg/L), the concentration units cancel and *K_c_* is dimensionless. The Gibbs free-energy change was calculated using Δ*G*° = −*RT*ln*K_c_*. The values of Δ*H*° and Δ*S*° were obtained from the slope (−Δ*H*°/*R*) and intercept (Δ*S*°/*R*), respectively, of the Van’t Hoff plot of ln*K_c_* versus 1/*T*, following the thermodynamic approach reported previously [62]. The gas constant *R* was taken as 8.314 J mol-1 K-1, and temperature was expressed in Kelvin. Values obtained in J/mol were divided by 1000 for reporting Δ*G*° and Δ*H*° in kJ/mol, while Δ*S*° was reported in kJ/(mol·K). *K_c_* was calculated separately at each experimental temperature.

## 4. Conclusions

This study developed a value-added rPET/sepiolite composite adsorbent for methylene blue (MB) removal from water using a solution-blending method. OFAT screening identified 10 wt% rPET, a TFA:DCM ratio of 3:7, and 0.25 g sepiolite as the selected formulation. A total blending time of 60 min was used, consisting of 30 min pre-blending followed by 30 min after sepiolite addition. This formulation gave the best adsorption performance among the compositions tested. The composite had an average particle size of 267.10 nm, which was lower than that of neat rPET and sepiolite. The reduction in particle size was consistent with smaller aggregates and better dispersion, which may improve the accessibility of the adsorbent surface.

The adsorption conditions were further optimised using a five-factor, three-level Box–Behnken design. The quadratic model showed high R^2^ (0.9892), adjusted R^2^ (0.9805), and predicted R^2^ (0.9568) values, with an adequate precision of 37.25. However, the significant lack-of-fit (F = 90.90, *p* < 0.0001) indicates that the model does not completely describe all systematic variation across the full experimental design space; therefore, its predictions were interpreted within the investigated factor ranges. ANOVA showed that adsorbent dosage had a particularly strong effect on MB removal. MB concentration, pH, and several interaction terms were also significant. Numerical optimisation identified 62.5 min contact time, 25.5 mg/L MB concentration, 2.55 g adsorbent per 50 mL, pH 7, and 55 °C as the selected operating conditions. Under these conditions, the model predicted 99.03% MB removal. Independent experimental validation gave 99.06% MB removal, showing close agreement with the predicted value at the selected conditions.

The kinetic, equilibrium, and thermodynamic results provided further information on the adsorption behaviour. The PSO model gave the best fit to the kinetic data, with R^2^ = 0.99996. However, this fit alone does not prove that chemisorption was the only mechanism involved. The Weber–Morris analysis showed that intraparticle diffusion was not the sole rate-controlling step, suggesting contributions from more than one mass transfer process. The equilibrium data were better represented by the Freundlich model (R^2^ = 0.9976), indicating adsorption on a heterogeneous surface with sites of different affinities. Thermodynamic analysis gave negative ΔG° values of approximately −8.93 to −9.24 kJ/mol over the investigated temperature range. The ΔH° value of −6.99 kJ/mol indicated an exothermic process, while the positive ΔS° value of +0.00653 kJ/(mol·K) indicated a small increase in disorder at the solid–liquid interface. The adsorbent also maintained more than 90% reusability efficiency over six adsorption–washing–reuse cycles, demonstrating short-term retention of adsorption performance following distilled-water washing.

Overall, the rPET/sepiolite composite showed high MB removal and good short-term reusability under the conditions studied. The results suggest that post-industrial PET can be converted into a clay-containing adsorbent for dye removal. Recent studies have similarly emphasised that adsorption performance should be assessed using adsorption capacity together with removal efficiency and operating conditions, particularly when considering practical wastewater treatment applications [67]. Future studies should also examine the adsorbent under more application-oriented reactor configurations and operating conditions, as demonstrated in recent dye removal studies using clay-based adsorbents [68]. Future equilibrium studies should also apply direct nonlinear isotherm fitting together with complementary error functions such as RMSE and *x*^2^ to provide a more rigorous assessment of model suitability. Recent clay-based dye adsorption studies also provide useful context for extending the equilibrium evaluation of such adsorbent systems [69]. Further work should evaluate post-cycle structural stability using morphological, chemical, and textural characterisation, together with testing in real textile wastewater and mixed-contaminant systems. Future work should quantify desorption efficiency by measuring MB released during the washing/desorption step, determine adsorbent mass recovery after each reuse cycle, and evaluate more effective regeneration/desorption protocols [70]. In particular, the environmental and economic implications of the TFA/DCM processing route, including solvent recovery and reuse, should be evaluated together with process economics and scale-up performance before practical application.

## Figures and Tables

**Table 1:** Particle size characteristics of rPET, sepiolite, and rPET/sepiolite samples.

Samples	Size (d.nm) ± 0.1	% Intensity
rPET	926.97	100.0
Sepiolite	1067.45	87.4
rPET/sepiolite	267.10	100.0

**Table 2:** The surface area, pore volume, and pore size of rPET, sepiolite, and rPET/sepiolite.

Sample	Surface Area (m^2^/g)	Pore Volume (cm^3^/g)	Pore Size (nm)
rPET	0.35	0.000826	14.72
Sepiolite	295.11	0.768224	20.01
rPET/sepiolite	0.67	0.003925	26.05

**Table 3:** Kinetic parameters for MB adsorption onto the rPET/sepiolite adsorbent.

Kinetic Models	Parameter	Value
Pseudo-first-order	q_e (exp)_(mg/g)	0.488
	q_e (cal)_ (mg/g)	0.791
	k_1_ (1/min)	0.03886
	R^2^	0.7466
Pseudo-second-order	q_e (exp)_(mg/g)	0.488
	q_e (cal)_ (mg/g)	0.492
	K_2_ (g/mg min)	5.17
	R^2^	0.99996
Intraparticle diffusion	K_id_ [mg/(g·min^0.5^)]	0.00521
	C (mg/g)	0.454
	R^2^	0.7452

**Table 4:** Linearised isotherm parameters for MB adsorption onto the rPET/sepiolite adsorbent.

Isotherm Models	Parameters	Value
Langmuir	qm (mg/g)	Non-physical estimate
Langmuir	K_L_ (L/mg)	Non-physical estimate
Langmuir	R^2^	0.9196
Freundlich	K_F_ [(mg/g)(L/mg)^1/n^]	0.0152
Freundlich	n	0.8525
Freundlich	R^2^	0.9976

**Table 5:** Thermodynamic parameters for MB adsorption onto the rPET/sepiolite adsorbent.

Adsorbent	Temperature, *T*(K)	ΔG° (kJ/mol)	ΔH° (kJ/mol)	ΔS° [kJ/(mol·K)]	R^2^
rPET/sepiolite	303.15 K	−8.93	−6.99	+0.00653	0.98
	328.15 K	−9.23	-	-	-
	353.15 K	−9.24	-	-	-

**Table 6:** One-factor-at-a-time (OFAT) formulation parameters and screening results for rPET/sepiolite composite adsorbent fabrication.

Parameter	Details
rPET Loading (wt%)	Evaluated conditions: 1, 5, 10, 15, 20 wt%. Fabrication method: rPET pellets dissolved in 3:7 TFA:DCM (*v*/*v*) under orbital shaking at 180 rpm for 24 h; sepiolite added (0.25 g); cast films dried, ground, sieved, and evaluated with MB dye. Evaluation metric: % MB removal (50 mL of 10 mg/L MB, 1.0 g adsorbent, 60 min, pH 7, 25 °C). Selected optimum: 10 wt% rPET. Scientific justification: Highest dye removal efficiency observed. Lower loadings (<10 wt%) formed mechanically fragile films, whereas higher loadings (>10 wt%) resulted in excessive dope viscosity that hindered clay dispersion.
Solvent Ratio (TFA:DCM, *v*/*v*)	Evaluated conditions: 9:1, 7:3, 5:5, 3:7, 1:9. Fabrication method: 10 wt% rPET dissolved at 400 rpm; 0.25 g sepiolite added incrementally; blended slurry cast onto glass Petri dishes, dried under vacuum, ground, and sieved. Evaluation metric: % MB removal (50 mL of 10 mg/L MB, 1.0 g adsorbent, 60 min, pH 7, 25 °C). Selected optimum: 3:7 TFA:DCM (*v*/*v*). Scientific justification: DCM acts as a volatile non-solvent pore-former inducing controlled phase separation. Excessive TFA (≥5:5) formed dense, non-porous skins, whereas 3:7 provided optimal porosity, film uniformity, and dye removal.
Sepiolite Content (g)	Evaluated conditions: 0.0, 0.10, 0.25, 0.70, 0.80 g. Fabrication method: 10 wt% rPET dissolved in 3:7 TFA:DCM; varying masses of sepiolite dispersed under high shear (500 rpm); composite cast, dried, and pulverised. Evaluation metric: % MB removal (50 mL of 10 mg/L MB, 1.0 g adsorbent, 60 min, pH 7, 25 °C). Selected optimum: 0.25 g sepiolite. Scientific justification: Provided the highest MB removal among the evaluated sepiolite loadings. Higher loadings (>0.25 g) caused clay agglomeration and channel blocking and reduced active surface accessibility.
Blending Duration (min)	Evaluated conditions: 15, 30, 45, 60, 90 min. Fabrication method: 10 wt% rPET dissolved in 3:7 solvent and mixed with 0.25 g sepiolite at 500 rpm for varying times prior to casting and drying. Evaluation metric: % MB removal (50 mL of 10 mg/L MB, 1.0 g adsorbent, 60 min, pH 7, 25 °C). Selected optimum: 60 min (30 min pre-dissolution + 30 min clay dispersion). Scientific justification: Provided the best adsorption performance among the evaluated blending durations.

**Table 7:** Levels of independent variables and experimental range.

	Coded and Actual Levels		
Independent Variables	−1	0	+1
Contact time (minutes)	5	62.5	120
MB Concentration (mg/L)	1	25.5	50
Adsorbent Dosage (g in 50 mL)	0.1	2.55	5
pH	2	7	12
Temperature (°C)	30	55	80

**Table 8:** Nonlinear kinetic model equations and parameters used for MB adsorption.

Models	Equations (Nonlinear Form)	Parameters	Equations
Kinetic pseudo-first-order	*q_t_* = *q_e_* [1 − *exp*(− *k*_1_*t*)]	k1 = rate constant	(9)
Pseudo-second-order	qt = k_2_q^2^_e_t/1 + k_2_q_e_t	K2 = rate constant	(10)
Intraparticle diffusion	qt = k_i_t^1/2^ + C	Ki = intraparticle diffusion rate constant C = intercept associated with the boundary layer effect (mg/g)	(11)

**Table 9:** Isotherm model equations used for MB adsorption.

Models Isotherm	Equations (Nonlinear Form)	Parameters	Equations
Langmuir	$q_{e}=\frac{q_{m}K_{L}C_{e}}{1+K_{L}C_{e}}$	qm = maximum monolayer adsorption capacity (mg/g) KL = Langmuir constant for adsorption energy	(14)
Freundlich	$q_{e}=K_{F}C_{e}^{1/n}$	n = Freundlich adsorption intensity KF = Freundlich adsorption capacity constant	(15)

## Data Availability

Data available online as Supplementary Materials; the remaining data can be requested from the corresponding author and will be provided upon reasonable request.

## Supplementary Materials

The following supporting information can be downloaded at https://www.mdpi.com/article/10.3390/ijms27198841/s1. References [58,71,72,73] are cited in Supplementary Materials.
