# Supplementary material for: Recycled PET/Sepiolite Composite Adsorbent for Methylene Blue Removal: Formulation, RSM Optimisation, Reusability, and Adsorption Mechanism
Source: Int J Mol Sci. 2026 Oct 3;27(19):8841. doi: 10.3390/ijms27198841 (PMC13665175; doi:10.3390/ijms27198841)
Supplement: Supplementary file 1 [file ijms-27-08841-s001.zip › ijms-4584128-supplementary.pdf]

Table S1. *Arrangement of BBD for the five factors and the response (actual and predicted).*

Run	Factors					Responses	
	A (minutes)	B (mg/L)	C (g)	D	E (°C)	Actual (%)	Predicted (%)
1	62.5	25.5	2.55	7	55	99.3	99.03
2	5	25.5	5	7	55	94.6	91.54
3	62.5	1	0.1	7	55	46.0	43.34
4	62.5	25.5	2.55	7	55	99.2	99.03
5	62.5	25.5	2.55	12	30	94.7	97.14
6	62.5	25.5	2.55	2	80	94.8	91.77
7	62.5	50	2.55	7	30	85.7	82.93
8	62.5	25.5	2.55	7	55	99.0	99.03
9	5	1	2.55	7	55	98.6	98.91
10	5	25.5	0.1	7	55	24.4	24.83
11	62.5	25.5	0.1	12	55	57.1	48.66
12	5	25.5	2.55	7	80	93.2	94.44
13	62.5	25.5	5	7	30	96.1	98.59
14	62.5	25.5	2.55	7	55	99.2	99.03
15	62.5	25.5	5	12	55	94.5	88.80
16	62.5	1	5	7	55	93.1	90.55
17	62.5	1	2.55	7	30	99.7	100.95
18	62.5	50	0.1	7	55	9.9	11.91
19	62.5	25.5	5	2	55	99.9	106.84
20	62.5	1	2.55	2	55	100.0	101.87
21	62.5	25.5	2.55	12	80	100.0	105.40
22	5	25.5	2.55	2	55	89.1	86.26
23	5	50	2.55	7	55	80.1	81.87
24	62.5	50	2.55	7	80	96.5	92.97
25	62.5	25.5	5	7	80	91.0	91.54
26	62.5	50	5	7	55	96.8	98.90
27	62.5	1	2.55	7	80	97.6	98.03
28	5	25.5	2.55	7	30	85.7	87.01

Run	Factors					Responses	
	A (minutes)	B (mg/L)	C (g)	D	E (°C)	Actual (%)	Predicted (%)
29	62.5	25.5	0.1	2	55	8.6	12.76
30	120	25.5	2.55	2	55	96.9	95.81
31	120	1	2.55	7	55	98.8	97.46
32	62.5	25.5	0.1	7	80	35.1	35.04
33	62.5	50	2.55	2	55	82.4	82.14
34	120	25.5	2.55	7	80	95.7	94.62
35	62.5	25.5	0.1	7	30	19.0	20.88
36	62.5	25.5	2.55	7	55	99.4	99.03
37	62.5	25.5	2.55	7	55	98.2	99.03
38	120	25.5	2.55	12	55	96.8	99.24
39	120	25.5	0.1	7	55	25.9	28.48
40	62.5	50	2.55	12	55	98.8	99.26
41	62.5	1	2.55	12	55	100.0	102.61
42	62.5	25.5	2.55	2	30	98.8	92.91
43	120	25.5	2.55	7	30	95.9	94.93
44	120	25.5	5	7	55	96.9	95.98
45	5	25.5	2.55	12	55	100.0	100.69
46	120	50	2.55	7	55	91.3	91.42

Table S2. *ANOVA results for the proposed model for the response.*

Source	Sum of Squares	df	Mean Square	F-value	p-value	VIF
**Model**	32478.04	20	1623.90	114.18	< 0.0001	significant
A-Time	65.57	1	65.57	4.61	0.0417	
B-Conc. MB	532.57	1	532.57	37.45	< 0.0001	
C-Adsorbent Dosage	18011.65	1	18011.65	1266.46	< 0.0001	
D-pH	318.89	1	318.89	22.42	< 0.0001	
E-Temp.	50.62	1	50.62	3.56	0.0709	

Source	Sum of Squares	df	Mean Square	F-value	p-value	VIF
AB	30.31	1	30.31	2.13	0.1568	
AC	0.1560	1	0.1560	0.0110	0.9174	
AD	30.25	1	30.25	2.13	0.1572	
AE	15.02	1	15.02	1.06	0.3140	
BC	395.61	1	395.61	27.82	< 0.0001	
BD	67.08	1	67.08	4.72	0.0396	
BE	41.99	1	41.99	2.95	0.0981	
CD	727.38	1	727.38	51.14	< 0.0001	
CE	112.36	1	112.36	7.90	0.0095	
DE	22.04	1	22.04	1.55	0.2247	
A^2	125.55	1	125.55	8.83	0.0065	
B^2	69.71	1	69.71	4.90	0.0362	
C^2	10710.01	1	10710.01	753.05	< 0.0001	
D^2	0.5995	1	0.5995	0.0421	0.8390	
E^2	54.06	1	54.06	3.80	0.0625	
Residual	355.55	25	14.22			
Cor Total	32833.59	45				
R^2						0.9892
Adjusted R^2						0.9805
Predicted R^2						0.9568
Adeq Precision						37.2544

Note: Lack-of-fit test: F = 90.90, $p < 0.0001$, indicating a statistically significant lack of fit.

Table S3. *Summary of rPET-based and sepiolite-based adsorbent studies on dye adsorption.*

Adsorbent	Preparation Method	Key Findings	Key Characteristics and Limitations	Reference
PET / Multi-walled Carbon Nanotubes (MWCNT)	Phase inversion	Removed 51.53% MB at 6 mg/L. Demonstrated moderate removal efficiency but limited porosity development.	The material achieved moderate MB removal under the investigated conditions and incorporated carbon nanotubes as the functional phase. The study did not investigate clay incorporation or systematic optimization of rPET formulation variables such as polymer content, solvent ratio, sepiolite loading, and blending time.	(S M Lee et al., 2023) [71]
Graphene from PET Waste	Pyrolysis + chemical exfoliation	High surface area (721 m²/g). Adsorption was reported to be spontaneous and endothermic.	The conversion route produced a high-surface-area carbon material but required high-temperature pyrolysis (~800 °C) followed by chemical exfoliation. The resulting fine graphene powder may also require	El Essawy et al., 2017 [58]

Adsorbent	Preparation Method	Key Findings	Key Characteristics and Limitations	Reference
			additional recovery or separation steps after adsorption.	
NiO / Sepiolite Composite	Impregnation with NiO nanoparticles	High adsorption performance; endothermic behaviour; performance enhanced by NiO modification.	NiO modification enhanced the adsorption performance of sepiolite but required an additional metal-oxide impregnation step. The system does not incorporate or valorize PET waste, and the use of Ni-containing materials may require consideration of metal stability and potential leaching during repeated use.	(S. Gao et al., 2022) [72]
Sonicated Sepiolite	Ultrasonic treatment	Ultrasonic treatment improved MB adsorption compared with natural sepiolite	Ultrasonic treatment improved the adsorption performance of sepiolite compared with untreated clay. However, the approach modifies the clay	Küncek and Şener, 2010 [73]

Adsorbent	Preparation Method	Key Findings	Key Characteristics and Limitations	Reference
			phase alone, does not incorporate a polymer or PET-derived component, and requires ultrasonic processing equipment.	
